# Against cortical reorganisation

**DOI:** 10.7554/eLife.84716

**Published:** 2023-11-21

**Authors:** Tamar R Makin, John W Krakauer

**Affiliations:** 1 MRC Cognition and Brain Sciences Unit, University of Cambridge Cambridge United Kingdom; 2 Department of Neuroscience, Johns Hopkins University School of Medicine Baltimore United States; 3 Department of Neurology, Johns Hopkins University School of Medicine Baltimore United States; 4 The Santa Fe Institute Santa Fe United States; Western University Canada; Donders Institute for Brain, Cognition and Behaviour Netherlands

**Keywords:** plasticity, rehabilitation, deprivation, stroke, amputation, blindness, deafness

## Abstract

Neurological insults, such as congenital blindness, deafness, amputation, and stroke, often result in surprising and impressive behavioural changes. Cortical reorganisation, which refers to preserved brain tissue taking on a new functional role, is often invoked to account for these behavioural changes. Here, we revisit many of the classical animal and patient cortical remapping studies that spawned this notion of reorganisation. We highlight empirical, methodological, and conceptual problems that call this notion into doubt. We argue that appeal to the idea of reorganisation is attributable in part to the way that cortical maps are empirically derived. Specifically, cortical maps are often defined based on oversimplified assumptions of ‘winner-takes-all’, which in turn leads to an erroneous interpretation of what it means when these maps appear to change. Conceptually, remapping is interpreted as a circuit receiving novel input and processing it in a way unrelated to its original function. This implies that neurons are either pluripotent enough to change what they are tuned to or that a circuit can change what it computes. Instead of reorganisation, we argue that remapping is more likely to occur due to potentiation of pre-existing architecture that already has the requisite representational and computational capacity pre-injury. This architecture can be facilitated via Hebbian and homeostatic plasticity mechanisms. Crucially, our revised framework proposes that opportunities for functional change are constrained throughout the lifespan by the underlying structural ‘blueprint’. At no period, including early in development, does the cortex offer structural opportunities for functional pluripotency. We conclude that reorganisation as a distinct form of cortical plasticity, ubiquitously evoked with words such as ‘take-over’’ and ‘rewiring’, does not exist.

## Introduction

Neuroscience as a field relies on the idea that all brains within a species are similar and that there are also strong invariances in cortical organisation across species. Indeed, the latter assumption is critical to the generalisability of findings in animal models. Even research on inter-individual differences in brain function and structure, which has been gaining popularity, assumes that small differences are riding on top of larger commonalities. That said, one of the most well-known facts about the nervous system is that it is capable of change through plasticity. Plasticity is a general term often used to describe how a neuron, circuit, or brain region *quantitatively* changes so that a new output occurs in response to the same input. From a physiological perspective, cortical plasticity is largely attributed to the strengthening and weakening of pre-existing synaptic connections based on experience (e.g. Hebbian plasticity; Buonomano and Merzenich, 1998). Crucially, plasticity is driven by behaviour and in turn changes behaviour. Plasticity is a life mechanism – it is the process that allows us to develop from infancy to adulthood, to acquire new skills, to pursue new interests, and to compensate for aging. In short, it is the set of physiological processes that allows us to adjust to changes in our environment, our bodies, and our brain. But even though each of us is as unique as a snowflake, with profoundly distinct life experiences, we all share very similar brain functional organisation. For example, the organisation of the sensorimotor homunculus is largely determined by the time we are born, despite the restricted environment that the uterus offers (Arcaro et al., 2019; Dall’Orso et al., 2018). In this framing, cortical organisation is largely pre-determined by genetics, and plasticity mechanisms can only fine-tune it through learning and practice.

This conservative view of cortical plasticity in shaping brain function seems to be challenged, however, by dramatic examples of behavioural recovery, driven by seemingly equally dramatic neural changes, after either natural or experimentally induced injury to the peripheral or central nervous system in animal models and humans. It seems that a more extreme or special form of plasticity must be invoked, in essence a process that can match or live up to the impressive return (or enhancement) of capacities observed in such clinical cases. The term reorganisation is used in much of neuroscience to describe dramatic change in function; it refers to a distinct plasticity mechanism that is triggered by these edge cases, for example, blindness, amputation, or stroke. Reorganisation in this conception refers to a change to local processing, due to a plasticity change in input-output dynamics, resulting in a novel *functional* output from a given cortical area, which is distinct from the functional capacity of that area’s wild type. Such *qualitative* changes in a region’s computations have frequently been inferred from changes in the spatial layout of cortical maps, determined either by neural recordings or by functional brain imaging. Accordingly, it is commonly held that “*cortical maps of adult animals are not constant, but dynamic. The cortex can preferentially allocate cortical area to represent the selected peripheral inputs”* (Buonomano and Merzenich, 1998), and that “*The remodelling of the functional architecture of cortex in response to nervous-system damage could … provide a basis for recovery of function”* (Flor et al., 1995). Some researchers have even suggested that: “*At birth, human cortical areas are cognitively pluripotent: capable of assuming a broad range of unrelated cognitive functions”* (Bedny, 2017). Thus, it needs to be said from the outset that the case against reorganisation is *not* just a semantic argument, a mere quibbling over how terms are used. The implication of reorganisation is that that the cortex is much less functionally organised and specialised than classical localised architecture would suggest.

We are in complete agreement that clinical cases that show dramatic enhancement and recovery are fascinating and have hugely important implications for thinking about the underlying organisation of the cortex in health and disease. However, in our own personal experience, much of the seminal work that putatively demonstrates reorganisation has been misinterpreted over the years. In our view, the term reorganisation, if it is to deserve the name, implies ‘remodelling of functional architecture’, that is – a distinct change in the local processing in an area that results in a novel functional role. As such, reorganisation assumes that experience can override the genetic blueprint of brain function, and core to this idea is the notion that the cortex has, to varying degrees across the lifespan, a latent pluripotency that can allow a given cortical area to have novel computations assigned to it. It is this pluripotency that allows a given brain area to ‘take over’ from the lost area that originally performed them due to profound changes in behavioural abilities and needs. Indeed, there is a direct intellectual and conceptual throughline between considerations of how the cortex responds to injury and discussions of multifunctionality of neural networks in health, with invocation of reorganisation in the former as support for non-specialisation in the latter. Essentially, this is just a continuation, in more sophisticated form, of a debate about localised versus distributed capacities in the brain that dates to the 19th century. Positing that a function is performed by a non-localised distributed cortical network, in our view, conceptually overlaps with those that advocate for cortex’s capacity for large-scale reorganisation.

The popular view of cortical reorganisation, as it should be apparent in the quotations above, is invoking more than just strengthening of existing inputs to an area (or outputs, in the case of motor reorganisation), or applying pre-existing computations to those strengthened inputs. It also demands that the new function of the reorganised area be accurately interpreted by the rest of the brain to guide behaviour. Passingham and colleagues introduced the concept ‘connectional fingerprint’ (Passingham et al., 2002), which posits that a cortical region’s function is defined by its extrinsic connections and intrinsic properties. In this context, reorganisation signifies a change in a region’s connectional fingerprint; a change we consider qualitative. Thus, reorganisation goes beyond traditional synaptic plasticity mechanisms, such as Hebbian and homeostatic, and necessitates broader architectural modifications within the neural network. These large-scale changes are frequently linked to phenomena such as the sprouting of thalamocortical axons (Buonomano and Merzenich, 1998) and the formation of new long-range intracortical horizontal connections (Gilbert and Li, 2012).

**Figure 1.**
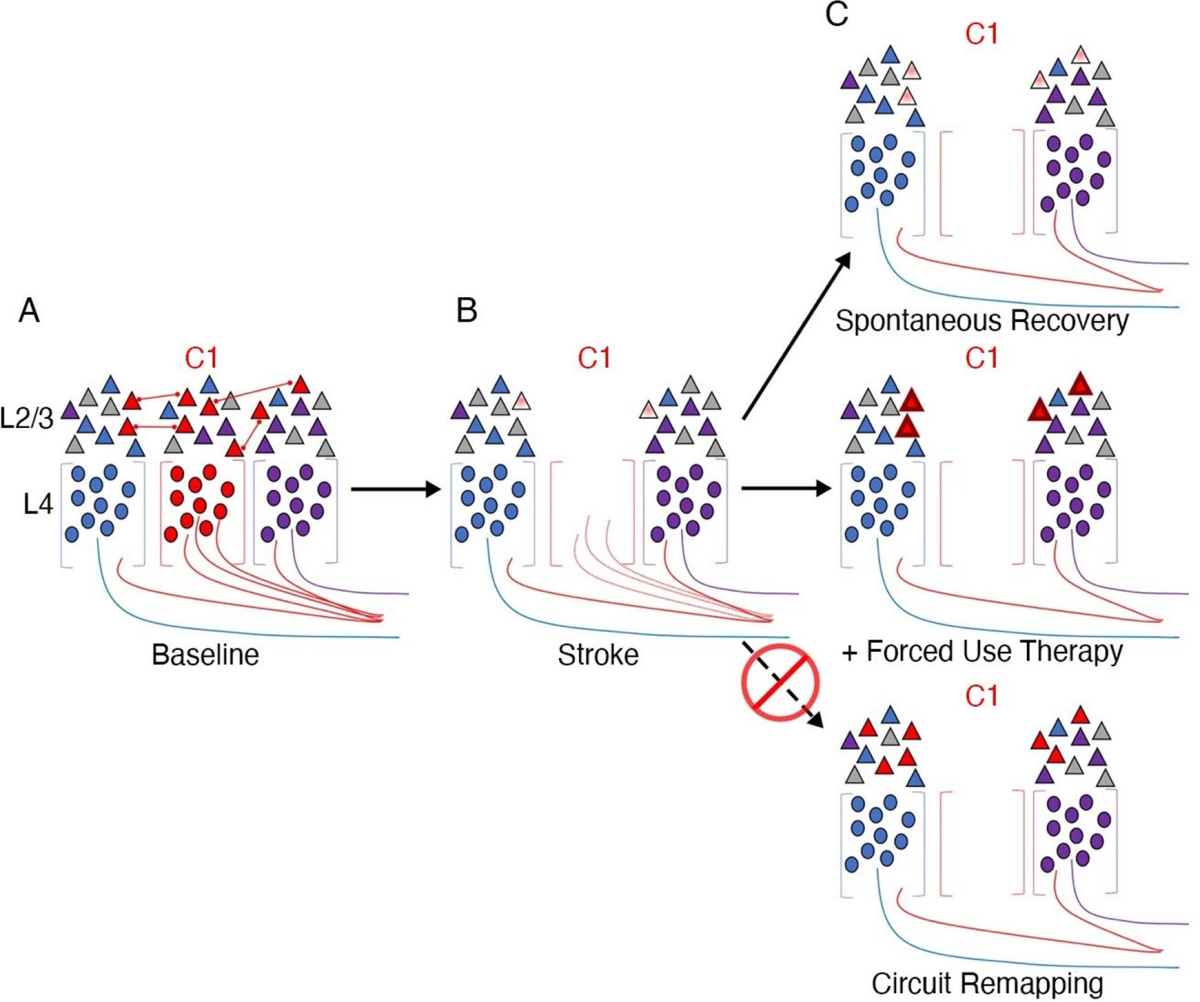
Stroke to the barrel cortex in mice unmasks existing latent functions but does not result in circuit reorganisation. An illustration of whisker remapping in somatosensory barrel cortex post-stroke. (**A**) Distribution of whisker-responsive neurons in L4 (circles, via thalamic input) and L2/4 (triangles, via intracortical inputs) in three adjacent barrels at baseline. Colours denote responsivity to a particular whisker (red = C1). Note that within a given barrel, L2/3 neurons are tuned to both the principal and surround whiskers. (**B**) After stroke targeting C1, the proportion of surround barrel neurons that are tuned to the C1 whisker attenuates, and their sensory-evoked responses are reduced (paler shading). (**C**) Spontaneous recovery (top panel) results in restoration of the proportion of surround barrel neurons tuned to the C1 whisker destroyed by stroke, but their sensory-evoked responses remain depressed, and there is no retuning of spared neurons to replace neurons lost to stroke. Forced use therapy (middle panel) after stroke restores and potentiates sensory-evoked responses in surround whisker neurons, but does not result in true circuit remapping with recruitment of new C1 whisker-responsive neurons (bottom panel). This figure was reproduced from Figure 7 from Zeiger et al., 2021.

As an illustrative example, let us consider a recent circuit-level analysis of changes in mouse primary sensory barrel cortex after a photothrombotic lesion destroying one of the barrels (Zeiger et al., 2021; Figure 1). In a typically developed mouse, each of the barrels would receive thalamic input from one principal whisker into layer 4. Following ablation of one barrel (C1), the surviving neighbouring cells are thought to be recruited to take over the native function of C1 (Murphy and Corbett, 2009). How does this happen? Reorganisation, as we interpret the literature, has two important categorical meanings. In the first case, it implies a change in the body part that a cell responds to or controls. In this exemplar case it would mean that neurons in the neighbouring barrels categorically change their tuning from their primary whisker input to the missing C1’s whisker. This could be achieved, for example, by sprouting of new corticothalamic connections. There was no evidence, however, of an increase in the population of C1 whisker-responsive neurons. Instead, cells in the neighbouring barrels that were already tuned to the C1 whisker were capable of being potentiated. Interestingly, tissue damage to C1 alone was not sufficient for this potentiation. Instead, upregulation of the responsiveness of the pre-existing C1 input was only achieved by behavioural pressure to increase its functional relevance (i.e. plucking of all other whiskers but the C1 native whisker). This example illustrates that a cortical area that has a mixed population of cells may favour one subset of cells over the others through upregulation, and thus appear to switch identity. However, this is a functional change at the level of its role in behaviour but the cortical area has not fundamentally reorganised structurally. The authors conclude: ‘Our findings are significant because they put into question the long-held remapping model of stroke recovery that has influenced stroke research for decades’. We agree with this conclusion and seek to show in our review article that this remapping misconception extends well beyond stroke recovery.

The second categorical form of the notion of reorganisation is that a cortical area can change its computational capacity. In the example we highlight here from Zeiger et al., 2021, this means that the cell in L2/3 that is now representing the C1 whisker has changed its connectional fingerprint. However, as demonstrated in Figure 1, this is not what happens (see also Rabinowitch et al., 2016, for an analogous example following sensory deprivation in nematodes). Perhaps this notion of changed computational capacity is better suited for instances of large-scale remapping – for example, a visual area can become either an auditory or a language area. A great deal of evidence, however, suggests that most of neocortex is multisensory (Ghazanfar and Schroeder, 2006). This is certainly true of higher-order association (multimodal) cortex and for a great deal of unisensory (unimodal) cortex (Schroeder and Foxe, 2005). This implies that multimodal cortex can process various forms of input in the wild type. In our view, what is called reorganisation is better interpreted as upregulation of a more general input-agnostic computational capacity that then favours one input over another. Once one rejects these two categorical versions of plasticity (reorganisation) then a much more interesting quantitative vista opens up, whereby functional recovery results from the combination of two processes: unmasking of latent pre-existing tuned cells in primary modality areas and redirection of general input-agnostic computational capacities in higher-order areas onto these unmasked primary modalities.

Here, we critically evaluate whether cortical map changes really indicate that a region has undergone reorganisation, and, regardless of whether it has or not, are such changes causally relevant to behaviour. These are two different questions, and we will address the scientific evidence through their two lenses. Rather than attempting to be exhaustive in our review of the literature, which would be impossible given the widespread acceptance of the notion of reorganisation, we will instead present, and critically discuss, a carefully chosen subset of examples. The examples chosen, in our view, home in on key methodological, empirical, and conceptual pitfalls that have led to contemporary confusion over the notion of reorganisation. We will begin by reviewing examples from early post-natal experience, as critical periods of development are widely assumed to offer a unique opportunity for reorganisation. We will next consider extreme circumstances of input loss, due to blindness, deafness, and amputation, as well as increased input due to extended practice or rehabilitation. We will also consider changes triggered by abrupt alterations to our body, such as hand transplantation and tool-use, as well as injuries occurring to the brain itself.

We will argue that mere changes in map features, reported across many canonical examples, do not meet the definition of reorganisation, that is, the appearance of a novel computational capacity in a given brain area. Instead, and despite the availability of synaptic plasticity mechanisms to modulate and facilitate existing brain architecture and dramatic changes in behaviour, cortical representation remains remarkably stable and invariant. This is because brain function is inherently grounded in its underlying structure – a functionally distinct territory will typically exhibit a characteristic cytoarchitectonic, histochemical and connectivity architecture and receive a specific set of inputs, which together determines its representational content. Representation is a very fraught term in neuroscience and philosophy (Krakauer, 2021). Here, we will use the term loosely to simply indicate a correlation between either aspects of the environment or the body and neural responses. This is usually determined by varying something in the world and finding a corresponding change in the brain.

We consider our revised framework to be more than just academic as it has direct implications for how we conceive and develop new approaches to neurorehabilitation. Specifically, we should shift from trying to induce (or reverse) reorganisation and instead take advantage of functionally relevant residual neural architecture with specific behavioural training and properly targeted physiological stimulation.

### Experimentally induced rewiring of retinal input in newborn ferrets

Mature cortical areas differ with respect to their molecular properties, histological organisation, intrinsic and extrinsic connectional fingerprint; differences which together determine a given brain area’s specific function. The unique identity of a given area is determined by genetic expression and is moderated by electrical activity over the course of early development (see Sur and Rubenstein, 2005, for review). It is in the earliest stages of brain development, when these organising features are still not fully expressed and cortical organisation is potentially under-determined, that pluripotency, if it exists, has its best chance to show itself. This phase of increased susceptibility to input in shaping the neural circuit is called a critical period (Levelt and Hübener, 2012). The critical period is thought to be enabled because plasticity brakes, such as inhibitory circuits and synaptic pruning, which normally preserve homeostatic balance, have not yet fully matured (Takesian and Hensch, 2013). As such, irregular input at this time (relative to a typically developing brain) should provide the ideal conditions for reorganisation to manifest.

**Figure 2.**
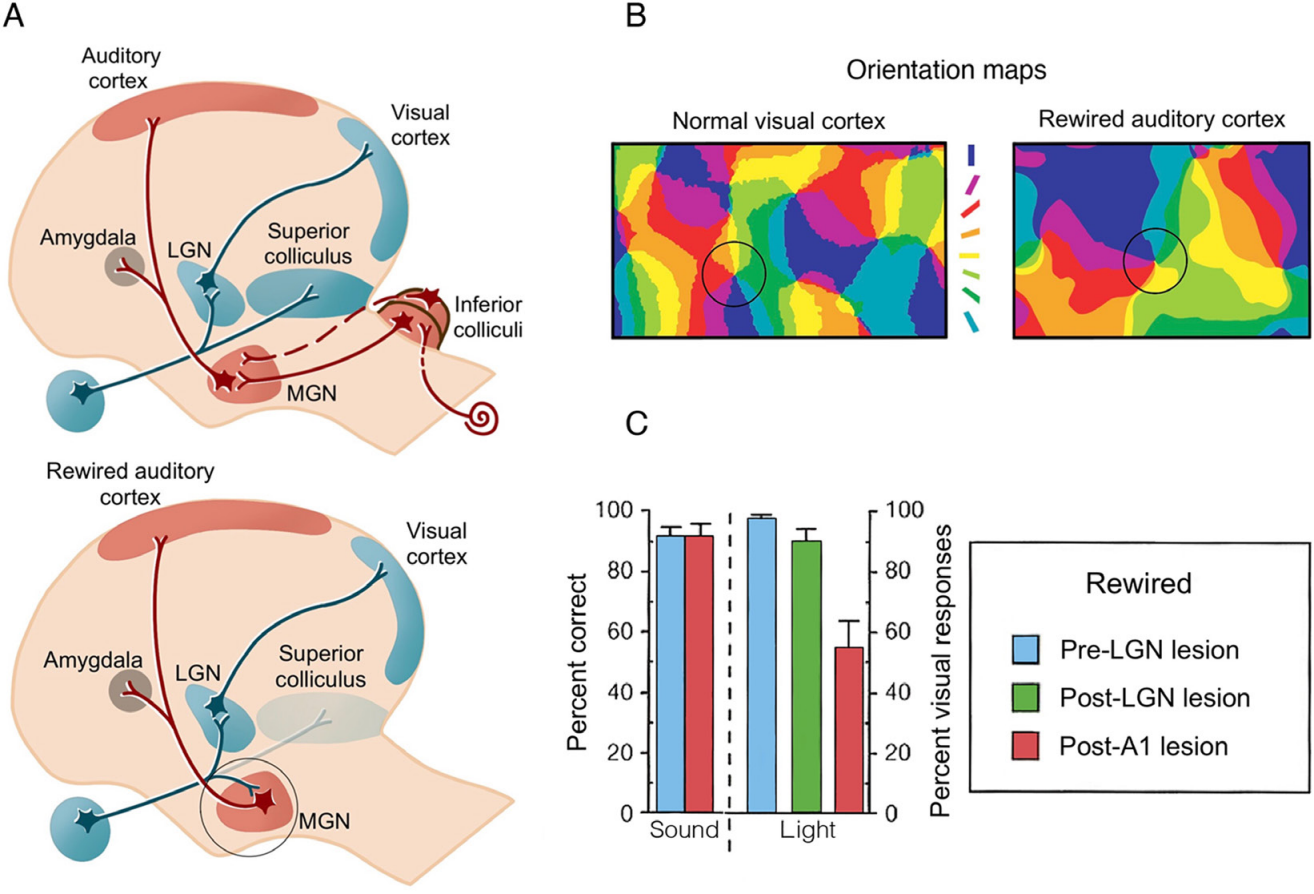
Artificial rewiring of retinal inputs into the ferret’s developing auditory thalamus. (**A**) Top: Normally, retinal information is routed to the visual cortex directly via the lateral geniculate nucleus (LGN), as well as via the superior colliculus, whereas auditory information is routed to auditory cortex (**A1**) via the inferior colliculus and the medial geniculate nucleus (MGN). Bottom: In a famous series of studies, retinal projections were diverted from the superior colliculus to the auditory MGN (highlighted in a circle), which directly connects to A1. Consequently, visual input is artificially fed onto an auditory pathway. (**B**) A winner-takes-all orientation selectivity map in normal visual cortex (V1) and in the rewired A1 showing similar visual functional organisation features. Qualitatively, the rewired A1 is showing patches of orientation-selective cells that is characteristic of V1. Orientation colour code is shown in the middle. (**C**) Sound versus light discrimination performance in a ferret undergoing unilateral visual rewiring. After visual rewiring and before lesioning (blue), both sound and light detection (contralateral to the rewired pathway) were high. A1 lesioning (red) resulted in profound reduction of light (but not sound) detection, whereas lesioning of the visual pathway (LGN/LP, green) did not significantly affect performance, providing causal evidence for the involvement of the rewired A1 in visual behaviour. (A–B) were adapted from Figure 4 from Sur and Rubenstein, 2005; (C) was adapted from Figure 2 from von Melchner et al., 2000.

Many studies that alter inputs to primary sensory cortices have been foundational for the formulation of the idea of cortical reorganisation. Perhaps the most striking example is the artificial re-routing of retinal inputs into the developing auditory thalamus (Sharma et al., 2000). In a series of technical tour de force studies, retinal projections in newborn ferrets (day 1) were diverted from the superior colliculus to the medial geniculate nucleus (MGN), a principle auditory thalamic nucleus that projects inputs to primary auditory cortex (A1) (Figure 2A). Consequently, when tested in adulthood, visual information triggered a patterned response in the rewired auditory cortex, such that distinct cortical patches within A1 showed selectivity to specific visual features, for example an orientation map which is typical of V1 (Figure 2B). Moreover, the horizontal connectivity profile in the rewired A1 was similar to V1 connectivity but quite distinct from the typical A1 structure. This is of interest, because the researchers did not directly manipulate cortical connections, but the changed inputs to thalamus nevertheless triggered an anatomical organisation reminiscent of visual cortex. These findings do suggest, early in development, that the organisation of a cortical territory can be shaped by changes in sensory input in combination with behavioural experience.

But is this true reorganisation in A1? According to our definition, we expect reorganisation in an area to fulfil three criteria: (i) appearance of a novel input (or output in motor cortex); (ii) emergence of a novel computation that is not present in the wild type, and (iii) that (i) and (ii) are functionally relevant. Here, the input was artificially rewired (i), and novel physiological responses were found, in the form of an orientation map (ii). Of course, caution must be taken when considering this example, first and foremost because the rewired thalamic input required an artificial procedure that would never occur through spontaneous plasticity mechanisms. And yet, we can still use this artificial scenario to ask whether the observed changes in A1 subserve a novel visual behaviour.

In a follow-up study, the rewired ferrets were trained to discriminatively respond to visual and auditory stimuli (von Melchner et al., 2000). The authors found that the ferrets were able to identify the stimuli as visual (and distinct from auditory) even while predominantly relying on inputs mediated by this rewired pathway, which was verified by lesioning the LGN. Most strikingly, this ‘visual’ behavioural performance was abolished when the rewired A1 was obliterated (Figure 2C). This elegant study provides a powerful demonstration of the causal involvement of a rewired, albeit artificially, region to drive visual behaviour and confirms that at least under these extraordinary circumstances, even a primary mammalian sensory area, in this case auditory cortex, is capable of some measure of sensory input-agnostic computation during post-natal development. Yet, the behavioural outcomes shown here – detecting a visual stimulus – doesn’t showcase orientation selectivity – the presumed new functional role ascribed to the rewired A1. So even in this dramatic artificial case, the results do not require appeal to a switch in the representational capacity of an area, but instead a physiological change due to a generic computation (signal detection) on a novel input. Indeed, the investigators themselves reach a similar conclusion in the original paper: “… intrinsic processing in primary auditory cortex may be similar in certain respects to that in primary visual cortex. This similarity might allow auditory cortex to process visual information; indeed, a parsimonious explanation of our results is that primary areas of sensory neocortex perform certain similar, stereotypical operations on input regardless of modality”. That is, the two brain areas share similar basic architecture, that is, feedforward thalamocortical inputs to layer IV that is then amplified by recurrent excitatory networks in cortex, and modulated by lateral inhibition, which underlies topographic representations (Rabinowitz et al., 2011; Tian et al., 2013). In this context, it is an open question whether the same result would have occurred if the visual input had been diverted into prefrontal cortex, which does not share the features of primary modality substrate. We should stress that these results are still seminal and point to a more granular idea of what kind of cortical plasticity is feasible given constraints. But based on this example it is likely that changes only occur in regions that already have significant overlapping similarities in their basic architecture. In the ferret case, an area with shared intrinsic properties to V1, namely A1, was artificially provided with the requisite extrinsic input. This seminal result, often cited as the locus classicus for reorganisation, instead laid the groundwork for an alternative explanation for many dramatic behavioural and physiological results. This alternative being the notion of input modality-agnostic computation.

### Recovery of language abilities after perinatal brain damage

The fact that auditory cortex is able to guide some basic visual behaviour when it is *engineered* to do so, that is, forced rewiring by a human experimentalist, does not inform us much on whether it occurs in more natural biological circumstances. Some of the most dramatic examples for remapping come from early in development. Accordingly, the existence of critical periods is often used in support of the idea that there is a special form of plasticity that is different from the garden-variety form that adults are left with. We now consider the functional consequences of profound alterations to brain architecture caused by perinatal stroke, which refers to strokes that occur between the middle of pregnancy and the first month of a new-born’s life. When brain damage to both grey and white matter is extensive, it will cause abnormal brain development. Based on the artificial rewiring study detailed above, we would expect that the developing brain should have some capacity to reorganise to best support the behavioural needs of these infants as they develop. But unfortunately, recovery from perinatal stroke is often poor, with most patients developing lifelong neurological disabilities, spanning motor, cognitive and other behavioural impairments (Dunbar and Kirton, 2018). This means that even if in early development, when one pathway (or a specific cortical region) is impaired, the functional role of this cortical territory is not spontaneously reassigned (or at least not successfully).

**Figure 3.**
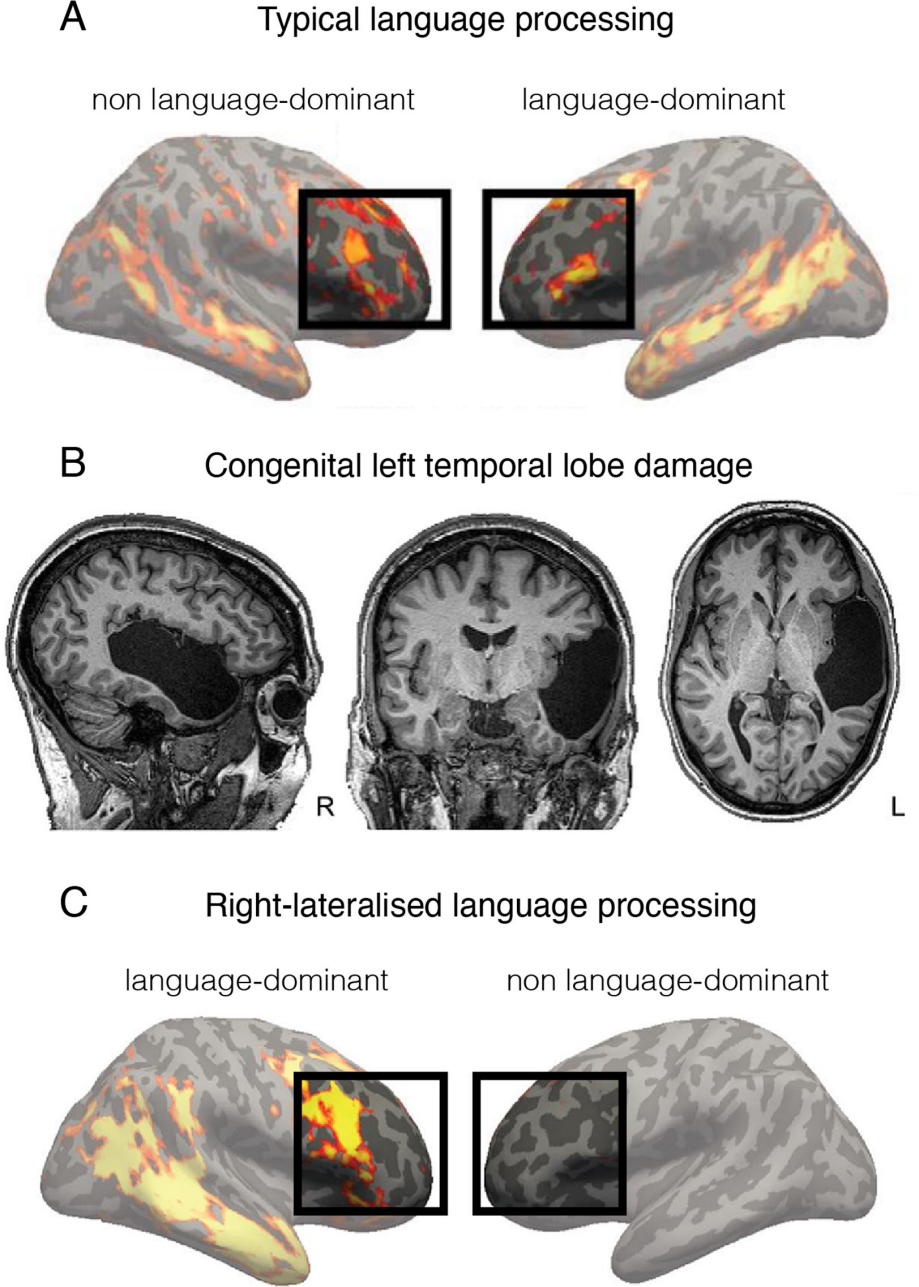
Language is processed bilaterally. (**A**) An example of a functional MRI (fMRI) activity map acquired during a language processing task (sentences versus nonwords contrast) in a typically developed individual. The prefrontal language areas are highlighted for emphasis. While the activity is stronger in the left hemisphere (commonly recognised as language-dominant), activity is also very clearly present in the homologous areas in the right (non language-dominant) hemisphere. (**B**) An individual born without a left temporal lobe. (**C**) In the absence of a left temporal lobe, language processing is shifted exclusively to the homologous temporal and frontal areas in the right hemisphere. But considering these same brain areas are already activated during language processing in controls (shown in A), it is unnecessary to invoke reorganisation as an underlying process. This figure is adapted from Figures 2a, 3a and b from Tuckute et al., 2021.

One potential exception relates to the neural mechanisms of language. Although language outcomes are often affected, some children with neonatal stroke show normal language performance (Fuentes et al., 2016). Functional MRI (fMRI) studies of children who sustained left hemisphere perinatal stroke found that (typically left dominant) language-related activation in inferior frontal cortex now occurred in anatomically identical areas in the right hemisphere (Raja Beharelle et al., 2010; Newport et al., 2022; Tillema et al., 2008; Tuckute et al., 2022; Figure 3). It is important to point out here that the changes from a predominantly left-lateralised to a right-lateralised activity profile for language are quantitative rather than categorical, as left-sided language processing reflects a dominance, rather than total disengagement of the right hemisphere. This is illustrated in Figure 3A, showing a typical participant performing a language localiser task. Indeed, other studies have reported an overall deficit in language development after perinatal stroke regardless of the side of the lesion (de Montferrand et al., 2019). That is, even children sustaining cortical damage to their right hemisphere showed impairments, indicating that the right hemisphere plays a causal role in language development.

As further evidence, some individuals show ambilateral or even right-lateral language processing even in the absence of brain damage (Joliot et al., 2016), clearly indicating that the right hemisphere has the functional capacity to process language. This is consistent with the notion that connectional homotopy is strongly reflected across hemispheres (Glasser et al., 2016). In a recent neurocomputational bilateral model, Chang and Lambon Ralph, 2020, showed that premorbidly, language processing already engages a distributed and largely bilateral network, albeit with a leftward asymmetry. They propose that across life, the right hemisphere can partially support language post lesion because it is not completely suppressed; instead, it has latent language capacities that already contribute to normal function. In support of this idea, multivoxel pattern analysis revealed that the pattern of activation in the right frontal cortex during auditory comprehension was predictive of subsequent language recovery in adults suffering from stroke (Saur et al., 2006).

Therefore, the interesting fMRI findings relating to right hemisphere language processing following early-development brain damage indicate that even when we can identify a change in the utilisation of resources to support a specific function, this change will be limited to spared circuitry that most closely mirrors the original connectional fingerprint. Indeed, this is what a homologous region *is*. This is strikingly demonstrated in Figure 3, showing similar engagement of the right hemisphere in a control participant with a left-lateralised language network (Figure 3A) and in a patient born without a left temporal lobe and hence doesn’t have a left hemisphere language network (Figure 3B–C).

Going back to our definition of reorganisation, requiring (i) novel input; (ii) novel computation, and (iii) novel connectional fingerprint to render (i) and (ii) is functionally relevant. The increase in right hemisphere lateralisation following left hemisphere lesions may not represent a full compensation, but rather a re-optimisation of the existing bilateral system, based on learning and plasticity mechanisms (Chang and Lambon Ralph, 2020). Therefore, reorganisation (as defined by us) is not necessary because the right hemisphere’s homologous regions already have latent shared capacities. These and other studies highlight the redundant computational capacity of multiple brain networks to potentially allow for optimisation of functional read-out of latent, or spare, processes (Stefaniak et al., 2020). In order to create better therapeutic approaches and maximise spontaneous recovery of language function after stroke, it is crucial to understand the mechanisms underlying the reweighing of computational contributions in existing regions and the role of both left and right hemispheres in these processes.

It thus appears that there is no strong evidence in the paediatric literature for true reassignment of brain resources due to perinatal stroke, that is, reorganisation, because the right homologous area already has latent shared capacity. It remains largely unknown what the precise critical period plasticity conditions are early in life that allow for bringing a homologous connectional fingerprint ‘on-line’ after injury (Werker and Hensch, 2015). In adults with left hemispheric stroke causing global aphasia, switching to the contralateral homologous language area, as seen after perinatal stroke and hemispherectomy in children, is also observed but recovery is much less dramatic (Karbe et al., 1998). Indeed, in adults, resting-state functional connectivity measures find clear statistical differences between the intrinsic connectivity patterns of language areas (Broca’s and Wernicke’s) in the left and right hemispheres (Nielsen et al., 2013). It is not clear whether its these differences or the presence of critical period (or both) that explains poor recovery in adults.

### Monocular deprivation in kittens

Many current assumptions about reorganisation can be traced back to Hubel and Wiesel’s seminal work on visual monocular deprivation. At the time, Hubel and Wiesel had been characterising how primary visual cortex (V1) is organised. As part of this exploration, they were interested in how input loss will impact the characteristic response profiles they observed in normal animals. For this purpose, the researchers occluded (e.g. sutured the eyelid) the right eye of new-born kittens (just before normal eye opening) for a period of several months (Wiesel and Hubel, 1963). At the time of this first study, they did not have a clear hypothesis as to what would happen to V1 organisation.

**Figure 4.**
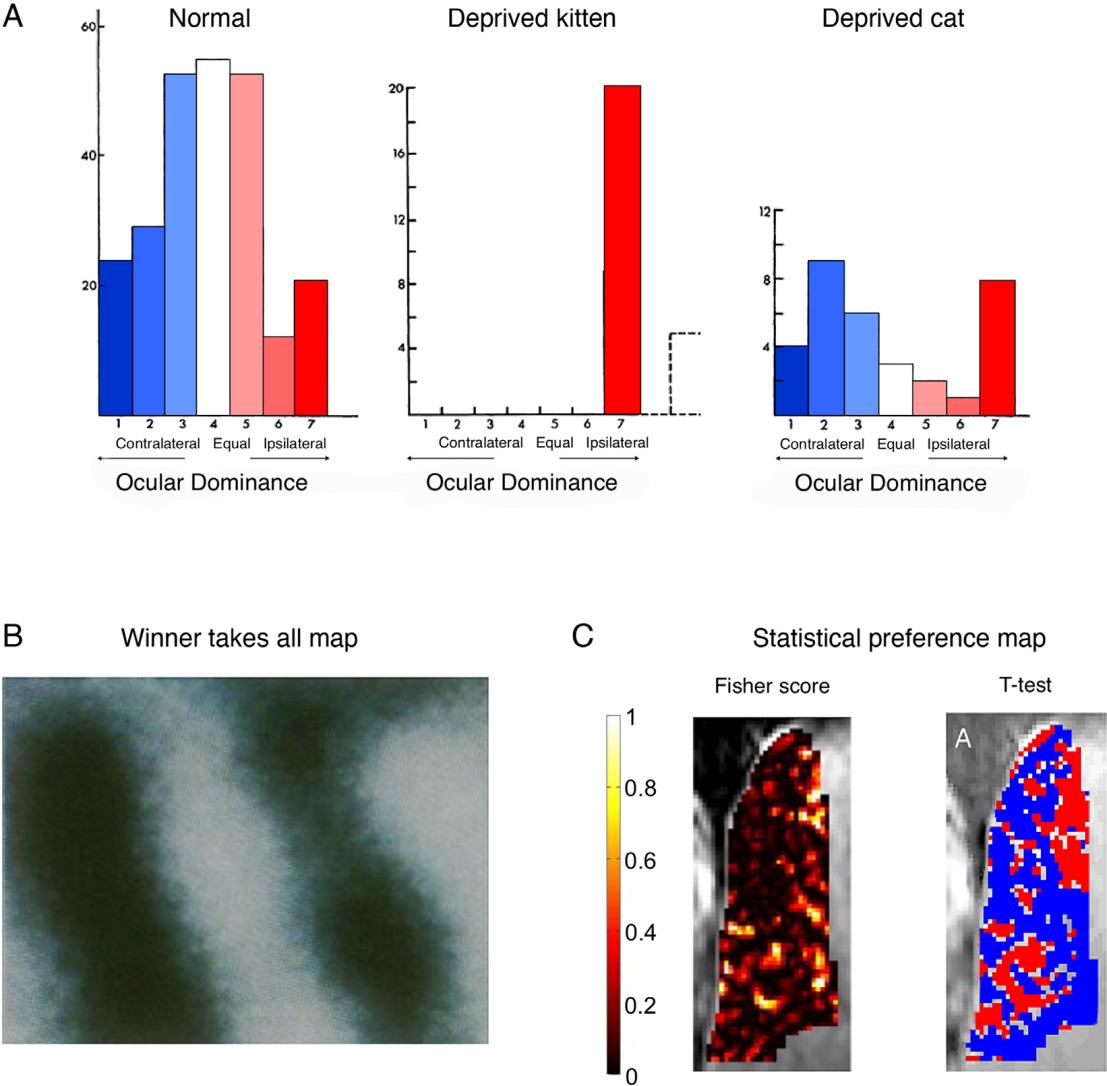
Cortical ocular dominance in primary visual cortex is not categorical. (**A**) Ocular dominance in the cats’ visual cortex, as originally reported by Hubel and Wiesel. In normally developed kittens, more cells show a preference to visual input from the contralateral eye (blue), as compared to the ipsilateral eye (red), as indicated by the slight leftward shift in the cells distribution. However, many cells only show weak, and even no preference to one eye versus the other, as indicated by a large number of cells in the middle of the distribution. Following ocular deprivation in early development of one eye, all cells show a strong preference to input from the open (ipsilateral) eye. But considering that most cells showed some response to the ipsilateral eye *independently of the* occlusion, reorganisation need not be invoked as an explanation of the post-occlusion responses. Monocular occlusion of the one eye in adult cats doesn’t result in a strong shift of dominance towards the spared (ipsilateral) eye. (**B**) A winner-takes-all map of ocular preference (acquired using optical imaging in a monkey). Dark bands represent columns dominated by input from the right eye, and the light bands represent columns dominated by input from the left eye resulting in the canonical binary (and highly misleading) map of ocular columns with crisp borders. (**C**) Alternative analyses (and colour scales) lead to very different depiction of ocular dominance maps. For example, consider the visualisation of a ultra-high (7 T) functional MRI (fMRI) dataset in a human, reporting the spatial distribution of ocular dominance. The Fisher score (left panel) highlights that most voxels do not strongly discriminate between the two eyes. However, after imposing a minimal statistical threshold (p<0.15; using a t-test relative to the other eye) and using binary red/blue labels (for the right/left eye, respectively), then the binary spatial columnar features emerge (right panel). We argue that the non-thresholded and non-binarised map provides a more accurate portrait of the functional attributes of ocular dominance. (A) is adapted from Figures 1, 3, 12 from Wiesel and Hubel, 1963; (B) was reproduced from Bartfeld and Grinvald, 1992. Copyright of National Academy of Sciences; (C) was reproduced from Shmuel et al., 2010.

When examining the visual abilities of the occluded eye once it has been reopened, the researchers described some very clumsy kittens – it was clear that visual perception of the reopened eye was profoundly impaired. When characterising the physiological responses in the formerly deprived (left) visual cortex, they found that the vast majority of V1 neurons responded to input exclusively arriving from the unaffected (left, ipsilateral) eye, that is, strong ocular dominance. When repeating the same occlusion procedure in an adult cat, ocular dominance was far less affected (Figure 4A).

Interestingly, Hubel and Wiesel, and others, did not initially interpret their results as evidence for plasticity, let alone reorganisation. At the time of these first studies, the organisational principle of ocular dominance was not established. In the ensuing years after Hubel and Wiesel’s work, the organisational feature of cortical ocular dominance has been strongly established, for example using optical imaging techniques (Bartfeld and Grinvald, 1992; Figure 4B) and is today considered a fundamental map feature of V1 organisation (Vidyasagar and Eysel, 2015). This later cementing of the phenomenon of ocular dominance columns allowed for a post hoc framing of the classical kitten study as an elegant textbook example of brain reorganisation – when input is locally deprived due to early life occlusion, then the deprived cortical columns are ‘taken over’ by the unaffected eye. Consequently, when reopening the eye, the neural circuitry is no longer available to adequately support visual processing from the reopened eye. This process only occurs during a critical period of development, while the brain is still sufficiently under-determined to allow for this pluripotent form of plasticity. However, once this one-eye ocular dominance has been established, the functional architecture is set, resulting in profound and long-term functional deficits to the occluded eye even after it had been reopened. Again, it needs to be asked whether the kitten study really supports this reorganisation story.

Mapping analyses tend to treat neurons (or voxels) with no selectivity similarly to those showing weak or artefactual activity, resulting in a misleading visualisation of the ocular map as binary. But, if the vast majority of neurons labelled as ‘right eye dominant’ already receive input from both eyes, there is no need to invoke reorganisation as a driving mechanism when identifying left-eye activity – the relevant input (from the left eye) was available all along. Physiologically, based on these original findings, there is no clear evidence for retuning of V1 neurons from one eye to the other eye. Later research tracking individual cells in the mouse visual cortex across episodes of monocular deprivation has successfully demonstrated that the input preference of single neurons shifts during deprivation towards the open eye, then returns to baseline tuning after the eye is opened (Rose et al., 2016). This is consistent with the interpretation that, in the absence of input from one eye, pre-existing receptive fields will undergo an increase in the gain of their responsiveness to input from the other eye. Interestingly, in the same paper, the authors note that functional network connectivity was stable despite the cell-level changes. These findings nicely mirror our introductory example from the barrel cortex (Figure 1), showing that a cortical area that has a mixed population of cells may favour one subset of cells over the other through upregulation, and thus appear to switch identity. However, the cortical area has not fundamentally reorganised structurally.

Importantly, there is no clear evidence from these studies that this gain modulation relates to a qualitative change in the cortical representation of unaffected eye. Reorganisation does not need to be invoked here because the input, computations, and behavioural capacity for the left eye were already present in the neuronal circuit. Instead, this elegant example should be taken as evidence for the increased capacity for brain plasticity in early development, meaning strengthening of weak, or even latent, connections to shape and narrow the receptive field.

To conclude, the quintessential textbook example used to argue for the brain’s capacity for reorganisation during the critical period of development is misleading. Given that many V1 neurons respond to inputs from both eyes (Figure 4A), the binary visualisation of ocular dominance ‘map’, based on the either/or idea of dominance, is wrong. That is to say, the sharp boundaries making up this map are the consequence of introducing this binary feature into the analysis itself. The idea of dominance plus its constructed visualisation through arbitrary thresholding thus reified reorganisation.

The series of studies led by Hubel and Wiesel on plasticity in ocular dominance columns following monocular deprivation inspired the widely popular treatment of amblyopia using an eye patch on the dominant eye of children, with limited success (Sengpiel, 2014). The rationale behind patching is that occluding the unaffected eye prevents ‘take-over’ of visual real estate in V1 of the amblyopic eye and thereby allows visual experience to promote recovery of visual acuity in that eye. But more recent work has demonstrated that the perceptual deficits that have been recorded following monocular deprivation in kittens can be reversed to a substantial extent by exposing the kitten to binocular vision (Kind et al., 2002). Behaviourally, although the conventional occlusion therapy for amblyopia has been shown to restore visual acuity in the amblyopic eye, it has rarely been shown to improve binocular function. We are not aware of any strong evidence to suggest that improvements following occlusion therapy are long-lasting and return of the deficit is common once treatment is completed (Bhola et al., 2006). There is also still no clear evidence that the unaffected eye – presumably benefiting from increased cortical resources – gains any benefits due to occlusion. These clinical findings support our view that reorganisation does not occur even during early development.

### Cross-modal plasticity in congenitally blind individuals

It could be argued that the case of monocular deprivation of a binocular cortical territory, as detailed in the section ‘Monocular deprivation in kittens’, is unsuitable for investigating true reorganisation. If the vast majority of neurons in V1 receive some input from both eyes, then there is no real need (and therefore opportunity) for reorganisation to occur in the first place. For this reason, binocular blindness provides an arguably better model for studying the capacity of the developing visual cortex for reorganisation. As visual animals, human’s cortex is dominated by visual input. Therefore, complete blindness, at least in theory, should free up a large amount of now supposedly unused cortex, which can now be reappropriated to support alternative modalities – hearing and touch. This idea is rooted in multiple behavioural observations, demonstrating heightened sensory acuity relative to sighted people for touch (Van Boven et al., 2000; Goldreich and Kanics, 2003; see also Figure 7) and sound (Röder et al., 1999). For example, many blind people are capable of discriminating millimeter differences in the spatial layout of raised dots (2.3 mm) while reading braille at an average speed of 7.5 characters per second (Legge et al., 1999); Some individuals navigate their way around physical barriers using acoustic cues (echolocation) alone (Stroffregen and Pittenger, 1995).

It has been proposed that these remarkable perceptual abilities are supported by cortical reorganisation. This is based on seminal studies suggesting that the primary visual cortex of blind people is activated during multiple tasks, such as when participants are reading braille (Sadato et al., 1996) or when they are listening to complex sounds (Poirier et al., 2006); a phenomenon termed cross-modal plasticity. The basic idea is that once ‘visual’ cortex becomes freed up from visual input, it is available to process other inputs via a range of different mechanisms, including both subcortical and long-range cortico-cortical connectivity alterations (Bavelier and Neville, 2002; Rauschecker, 1995). According to this proposition, the ‘visual’ cortex of the blind receives novel input, relative to the wild type (in accordance with our first criterion for reorganisation). It has further been proposed that these additional resources in ‘visual’ cortex allow blind individuals to develop their heightened sensory abilities (compatible with our third criterion for reorganisation). Later work also suggested correlations between behavioural performance and brain activity in primary visual cortex during non-sensory cognitive tasks, such as language, numerical processing, and memory (Amedi et al., 2003; Bedny et al., 2011; Kanjlia et al., 2016) (see Fine and Park, 2018, for a critical review of the underlying evidence). This last, and most radical, interpretation suggests that V1 is capable of truly novel computation (our second criterion for reorganisation), resulting in qualitatively different brain function not present in sighted individuals.

But does the evidence support the reappropriation of V1 to become, say, S1? This assertion cannot be made based on a simple observation of increased net activity in the visual cortex during the performance of non-visual tasks. This is because the increased activity observed in blind individuals could be a case of unmasking or gain modulation, that is, input responsiveness that is already there (and therefore also present in the wild type) but simply increased. This could be the consequence of reduced inhibition due to the congenital cortical deprivation (Butler and Lomber, 2013; Hahamy et al., 2017), or even homeostatic plasticity mechanisms – since the visual input is missing, the brain might over-express the (normally latent) non-visual inputs in order to achieve electric stability (see Muret and Makin, 2021, for a related discussion). In other words, the observation of increased activity for non-visual tasks in visual cortex of the blind doesn’t imply that this input is novel.

Crucially, homeostatic mechanisms could trigger substantial changes in net activity without requiring any changes to the functional read-out of a given area. Therefore, to support a claim of functional reorganisation, that is, novel local processing that supports novel behaviour, causal evidence is needed. In humans, a handful of TMS studies have reported that when processing in visual cortex of blind individuals was disrupted, perceptual abilities were also impaired. For example, TMS applied to the occipital pole doubled the errors made during Braille reading, relative to ‘air’ TMS (Cohen et al., 1997). Notably, the disruptive impact of the TMS mid-occipital stimulation was qualitatively greater than the impact of TMS stimulation over the sensorimotor (reading) hand territory itself. This raises a red flag, as any presumed reorganisation is not likely to have completely taken over the functional role of S1 in tactile processing: the contribution made by the visual cortex should not be *greater* than that of S1. TMS over V1 has since been shown to impair verbal semantic processing in blind individuals (Amedi et al., 2004), indicating that the disruptions in Braille reading may not be sensory at all. However, here, too, caution is needed. Considering that repetitive TMS will propagate its effects beyond the stimulation site (Bestmann et al., 2008), conclusive causal evidence in humans for the specific role of low-level visual cortex in supporting perceptual or cognitive abilities is still missing. Indeed, as we elaborate next, from the perspective of the ensuing 20 years, it appears that this initial account of cross-modal plasticity in V1 has not withstood the test of time.

Much work over ensuing years has been dedicated to careful scrutiny of initial claims of cross-modal plasticity in V1 (see Voss, 2019, for a comprehensive review). First, it has been established that cross-modal effects are mostly restricted to higher-order visual cortex, for example, lateral and ventral occipitotemporal cortex (see Figure 5 in Fine and Park, 2018). This is important because these areas have been suggested to be polymodal even in sighted individuals. In other words, they might not be strictly visual in the first place. For example, the human middle temporal complex (hMT+) has been reported to process tactile (Beauchamp et al., 2009) and auditory (Dormal et al., 2016) information in sighted individuals, as demonstrated using brain decoding studies. Indeed, after sighted people were trained to read Braille, they – too – activated the ‘visual’ word form area when reading Braille with their fingers (Siuda-Krzywicka et al., 2016). Unmasking of latent capacity that is present in sighted individuals is a more plausible mechanism than positing a qualitative change in a visual area to a tactile one. As to high-level cognitive processing in V1, to date there is no decisive evidence for such reorganisation in blind individuals. This is because the experimental paradigms aimed at studying complex non-visual tasks are riddled with confounds, leaving many alternative explanations open (see Fine and Park, 2018, for a related discussion).

**Figure 5.**
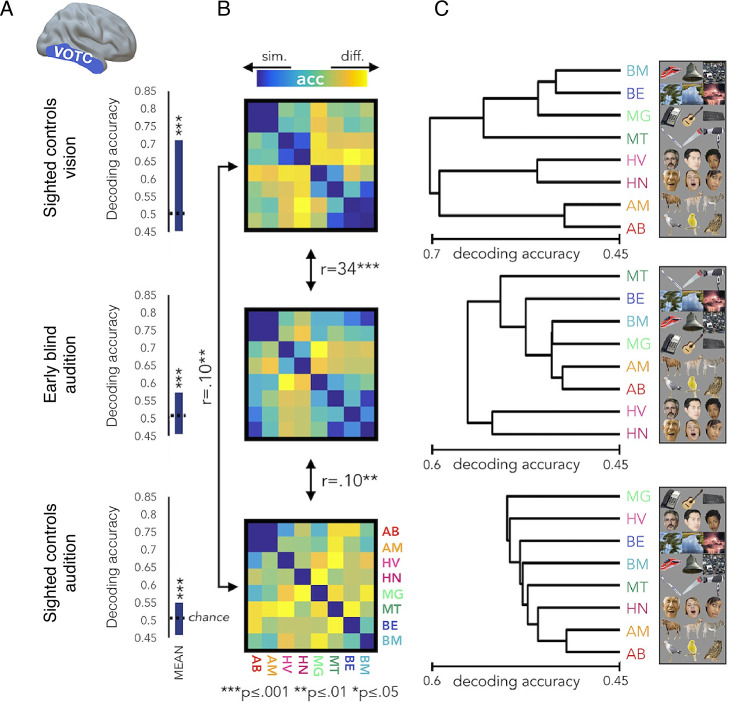
The representational structure of natural object categories in ventral occipitotemporal cortex is invariant between sighted and blind participants. The multivoxel representational structure across multiple natural object categories was studies in the visual domain in sighted controls, and compared with auditory representational structure found in the ‘visual’ cortex of early blind individuals, as well as sighted controls. The region of interest in the ventral occipitotemporal cortex (VOTC) used for the multivoxel pattern analysis is shown in the left. (**A**) Averaged binary decoding of functional MRI (fMRI) responses across all natural object category pairs. As expected, decoder accuracy was highest in the visual modality (top). However, for both groups, auditory information content for discriminating across object categories is also available in visual cortex, as indicated in significant above-chance decoding accuracy. This suggests that the cross-modal effects seen in blind individuals (middle) are already present, to some extent, in the sighted (bottom). (**B**) Representational dissimilarity matrices, showing binary decoding accuracy for each of the category pairs, for the different groups and stimulus modalities. (**C**) The binary cross-category decoding is plotted here as dendrograms reflecting hierarchical clustering, to allow an intuitive visualisation of the representational structure of categories in VOTC. The auditory representational structure in the blind individuals is more similar to the typical visual structure found in sighted controls. This is demonstrated in the greater correlation between the inter-category dissimilarity matrices for auditory categories in the blind and visual categories in the sighted. This is also portrayed in the more similar hierarchical clustering, as visualised in the dendrograms. This provides an elegant demonstration that cross-modal processing is confounded by the local representational features of this region, such that the underlying functional processing is relatively invariant. Therefore, the specific functional organisation of a given ‘visual’ area is maintained even if it is now driven by an auditory input – a principle termed ‘domain specificity’. Acc = decoder accuracy. This figure is adapted from Figure 4 from Mattioni et al., 2020.

One might argue that even if the input itself is not strictly novel, if the local processing of this input leads to a qualitatively distinct function, this might qualify as an anatomically restricted form of reorganisation. However, it has become increasingly clear that the putative cross-modal processing in higher-order visual cortex is not novel, or even general, as originally observed. Instead, it has been established that cross-modal processing is highly constrained by a pre-determined function, such that the specific computations of a given ‘visual’ area are maintained even if it is now driven by an input from another sensory modality (tactile, auditory) – a phenomenon termed ‘domain specificity’. For example, the extrastriate body area in sighted persons has been implicated in visual processing of body parts (Downing et al., 2001). Accordingly, in blind individuals, this body-selective region is specifically activated during recognition of body parts and body postures through touch (Kitada et al., 2014) or auditory cues (Striem-Amit and Amedi, 2014). Specifically, blind and sighted individuals haptically discriminating moulds of hand shapes and inanimate objects demonstrated preferential responses to hands, regardless of sensory modality or visual experience (Kitada et al., 2014). Another study demonstrated that the EBA in congenitally blind individuals is sensitive to full-body shapes conveyed through a sensory substitution device after prolonged intensive training (Striem-Amit and Amedi, 2014). So, the primary difference between occipital processes in blind and sighted individuals is their input modality. The use of more sophisticated analysis techniques, such as multivoxel pattern analysis, allows further characterisation of the representations responsible for the increased activity found in more classical studies by assessing the information content in a ‘reorganised’ brain area (see Box 1). For example, as we show in Figure 5, the representational structure of various object categories in the ventral occipitotemporal cortex, mediated either via sounds (in blind individuals) or vision (in sighted individuals), is similar (Mattioni et al., 2020). Note that in this example, some auditory information can also be extracted in the visual cortex of sighted people, again re-emphasising the idea that the cross-modal effects seen in blind people are attributable to representational structure already present in the sighted (see Rauschecker and Harris, 1983, for a similar observation). Therefore, while striking, cross-modal reorganisation is a lot tamer than originally advocated, and might be better characterised in terms of supra-modal functional organisation where the same abstract representation is encoded, independently of the specific sensory input (Castaldi et al., 2020). Importantly, under this framework, plastic changes are constrained by pre-existing neural scaffolding, both in terms of input, local processing, and read-out. In other words, the evidence better fits an account of plasticity (quantitative changes to existing circuitry) rather than one of reorganisation (qualitative changes to create novel circuitry).

Box 1. **Studying remapping using multivariate pattern analysis**

Historically, to infer changes to the representational features of a given brain area, cognitive neuroscientists have characterised univariate trends in response across a population, such as the spatial spread of units showing a similar feature preference, or the average activity level in an area across individual units. But let us consider a state-like change in population net activity, for example, due to aberrant inputs, disinhibition, cortical degeneration, or changed upstream processing. These changes will profoundly impact univariate outcome measures. Common to these mechanisms is that net activity gains/drops do not necessarily entail changed functional processing in that brain area that will necessarily impact the underlying representation. Conversely, local functional processing features can vary without impacting univariate measures such as average activity, especially when we consider the gross resolution of fMRI.

Alternative analysis tools are becoming increasingly popular for mining richer information of the processing underlying activity in a given brain cortex. Rather than looking for commonalities in activity between neighbouring voxels, multivariate techniques assess the stability of activity patterns across repetitions and stimuli. The underlying assumption is that subtle yet consistent variations in individual unit responses from one another may provide valuable information about the brain’s ability to process the relevant representational feature. Because these techniques consult with all voxels separately, they allows us to gather more information than most traditional approach that summarise one or two dimensions of the univariate response profile at a time. Therefore, multivariate analysis are sensitive to more subtle changes, that wouldn’t necessarily meet ‘threshold’ criteria in the traditional univariate approach. Previous fMRI multivariate studies identified representational features that we know exist in the underlying tissue, for example orientation dominance in visual cortex, that cannot be directly identified with traditional univariate analysis.

Representational similarity analysis (RSA) is a multivariate technique designed to determine how separate or distinct one activity pattern is to another. RSA allows us to ask not only if new information is available in a given brain area (i), but also whether this new information is structured consistently with known representational principles, for example related to behaviour (ii). (i) Inter-stimulus (dis)similarity (typically quantified using cross-validated Mahalanobis distances) indicates if there is sufficient representational information content to distinguish between two features, akin to a classifier. While univariate activity changes are likely to be identified by a dissimilarity analysis (and may also affect the distance measure indirectly, due to changes to SNR), RSA distances can also identify differences that are not reflected in the overall activity profile, because it essentially looks at the correlation between two patterns. In the context of reorganisation research, if a given area can reliably differentiate between new stimuli features to which it was not sensitive in the wild type (e.g. the deprived visual cortex differentiates auditory tones), then we can propose that a new computation has been formed. However, when using dissimilarity to infer a change in the representation (or any parallel measure, such as classification accuracy), there is always a concern that the differences that have been statistically identified are not necessarily functionally relevant. This is known as a feature fallacy – feature sets may be useful tools to describe distributions of activity profiles, but they do not carry a special significance in themselves. The feature fallacy becomes all the more relevant when we use computations that are relatively abstract from the underlying physiology, such as RSA. Moreover, as the multivariate distances may be influenced by to univariate activity changes, they too are malleable to some of the same risks we highlighted above. Therefore, we think that differences in information content should be interpreted with caution. (ii) The inter-distances representational structure (typically quantified using a correlation or a more formal model comparison) has the potential to provide a much stronger hypothesis for functional change. This is because the representational structure details an intricate framework of how multiple representational features should juxtapose relative to each other, as demonstrated in the representational dissimilarity matrix in Figure 5. By quantifying and characterising brain function beyond the spatial attributes of activity maps, while providing a more precise model for how information content varies across stimuli, we believe RSA provides an arguably better tool for assessing functional reorganisation.

Perhaps the most striking counterargument for the concept of cross-modal reorganisation in the congenitally blind visual cortex is the fascinating case of sight restoration in children as old as 12, by surgical removal of congenital dense cataracts (see Singh et al., 2018, for review). If the visual cortex is re-appropriated to support new functions, then it follows that restoration of visual input will be futile (or will at least require substantial reversal of reorganisation). But this is not the case. Not only are the children immediately able to perceive some visual information, they show susceptibility to visual illusions, such as the Ponzo illusion (where the lines further down the track are perceived as shorter, due to the angle of the tracks) (Gandhi et al., 2015; Singh et al., 2018). It is possible that sensory restoration is aided by an underlying residual input – perhaps these children were not completely blind after all, allowing for some residual input to support typical development of neural circuits. This confound was nicely addressed in a recent study in fish, which demonstrated that functional visual circuits emerge even in the absence of developmental activity (Barabási et al., 2022). These studies provide very compelling evidence that the functional organisation of sensory cortex is not substantially altered despite even profound and long-lasting deprivation.

### Cross-modal plasticity in the deaf

An arguably more sophisticated model for studying the plasticity potential of deprived sensory cortex involves research on visual processing in the auditory cortex of the white deaf cat, which is born deaf due to genetically triggered loss of inner and outer hair cells (Kral and Lomber, 2015). Except for deafness, these cats otherwise behave normally, with no detectable somatosensory, motor, or visual deficits. In fact, some visual abilities of these cats are superior to hearing cats, providing an interesting opportunity to determine whether these visual perceptual abilities are attributable to cross-modal plasticity in the cat’s deprived auditory cortex.

**Figure 6.**
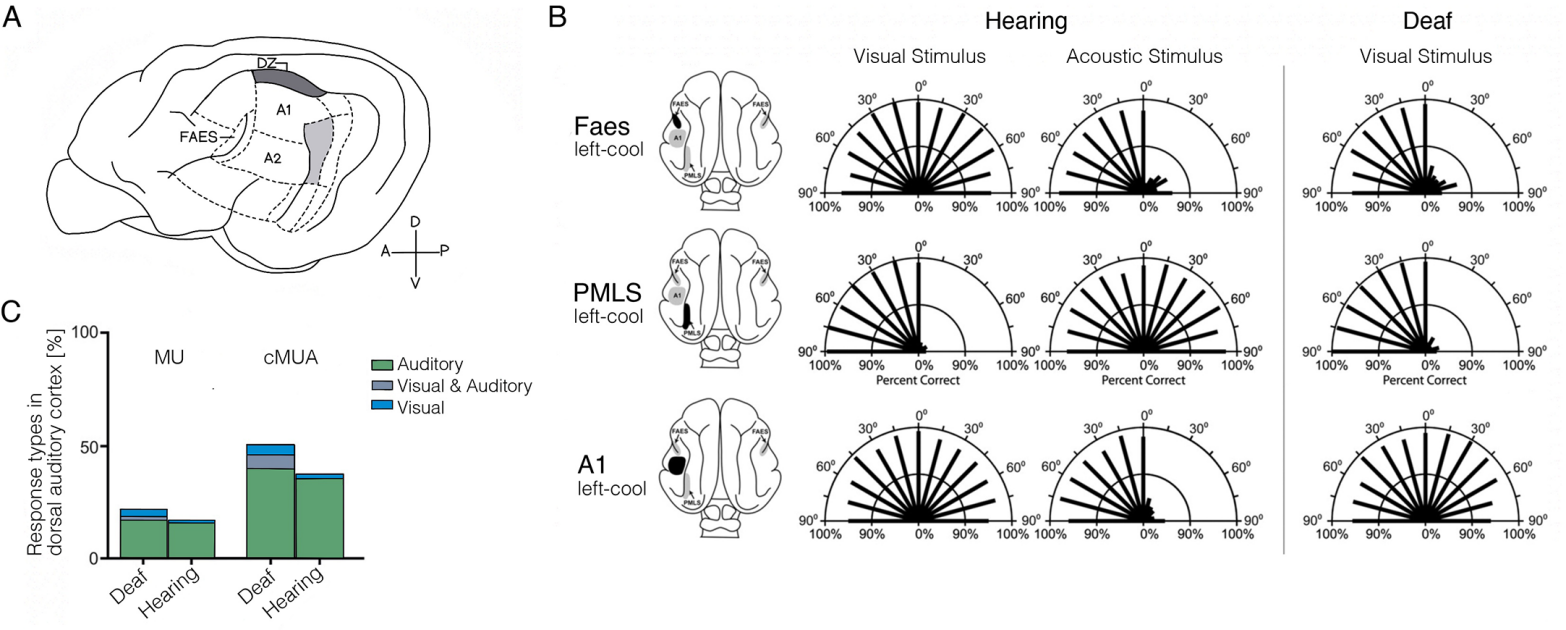
Cross-modal visual processing in higher-order auditory cortex has limited functional impact. (**A**) Lateral view of the cat cortex highlighting the locations of the higher-order auditory areas posterior auditory field (PAF), dorsal zone (DZ), auditory field of the anterior ectosylvian sulcus (FAES), as well as the low-level area A1. (**B**) Orienting responses to visual or acoustic stimuli during reversible cooling deactivation in hearing and deaf cats. Unilateral cooling of the auditory area FAES results in hearing, but not visual, contralateral deﬁcits in hearing cats, and induces contralateral visual orienting deﬁcits in deaf cats. Unilateral deactivation of the visual posteromedial lateral suprasylvian area (PMLS) results in contralateral visual orienting deﬁcits in both hearing and deaf cats, but does not affect acoustic orienting. Unilateral deactivation of A1 results in contralateral acoustic orienting deﬁcits in hearing cats, but has no effect on visual orienting in early-deaf cats. (**C**) Multiunit (MU; thresholded for spike amplitude) and continuous multiunit activity (cMUA; non-thresholded) recorded in DZ of congenitally deaf and hearing adult cats following a cochlear implant. Proportion of auditory (green), visual (blue), and visuo-auditory (grey) responses were recorded. Using both measures, auditory responses (green) are well matched between the two groups, whereas visual responses (blue), while present in the hearing cats, are more prominent in the deaf cats. (A) is adapted from Figure 5 from Butler et al., 2015; (B) is adapted from Meredith et al., 2011; (C) is reproduced from Figure 5C from Land et al., 2016.

The first study looking at the causal role of auditory cortex in mediating visual perception reported by Lomber et al., 2010. They first compared visual abilities in three deaf and three hearing adult cats with an extensive battery of visual tasks. They identified superior visual performance by the deaf cats in two instances: localisation in the far periphery (eccentricity ≥ 60 degrees) and motion detection. Thus, rather than a generalised overall improvement in visual performance, it seems that only speciﬁc visual abilities improved, ones that could be more general and apply across sensory modalities. The increased visual abilities were suggested to be supported by processing in *higher-order* auditory cortex, as demonstrated using bilateral cooling to selectively deactivate portions of auditory cortex that are thought to comprise the ‘where’ pathway, across which spatial and motion information are likely processed. Specifically, the researchers found that visual localisation was selectively degraded in the deaf cats when the posterior auditory field (Figure 6A), normally involved in acoustic localisation, was deactivated. Thus, a localisation area in auditory cortex contributed to visual localisation, which suggests that computations for localisation might be modality invariant rather than positing a switch in modality processing itself. Cooling of the dorsal zone of the auditory cortex (DZ; Figure 6A), also associated with auditory spatial processing (Stecker et al., 2005), specifically degraded the enhanced motion detection shown by the deaf cats. As we detail below, DZ is known to contain neurons with visual properties even in the wild type cat brain. Therefore, this research, too, advocates for the notion that local input, processing, and behaviourally relevant read-out for cross-modal plasticity are constrained by pre-existing neural scaffolding. In both cooling instances, the deaf cats’ enhanced visual performance was returned to similar levels as the hearing cats – that is to say, visual-specific processing was not affected. Importantly, visual performance of the hearing cats was not negatively affected by the selective deactivation of either of these areas, ruling out a trivial knock-on effect of the cooling to visual areas.

Another study in three early-deafened cats (at 8 days) demonstrated a similar effect: cooling of the auditory field of the anterior ectosylvian sulcus (FAES; Figure 6A) – normally involved in localisation of sounds – resulted in deficits in visual localisation (Meredith et al., 2011). Here, visual performance at baseline was similar between hearing and deaf cats. As shown in Figure 6B, unilateral auditory cortex deactivation eliminated the deaf cats’ visual performance almost entirely on the contralateral side, without an effect on visual performance in the hearing cats. In fact, the deficit on visual localisation in the deaf cats (Figure 6B, top-right) was similar to the deficits induced in auditory performance using the same procedure in the hearing cats (top-left). So here again – deactivation of specific sub-regions of higher-order auditory cortex impairs those more general perceptual abilities that can be re-assigned from the auditory modality to the visual one. In this study, the researchers also characterised the visual receptive field properties of single neurons in FAES, which were comparable to those in the same area in the hearing (control) cats, though they were more numerous (see also Korte and Rauschecker, 1993; Rauschecker, 1996). This nicely converges with the observations we highlight in Figure 5, where we find evidence for similar cross-modal processing in the typical sensory cortex, with the cross-modal input enhanced in the deprived cortex.

Is it possible that the auditory cortex of the deaf cats is now supporting low-level visual processing? To test this idea, the researchers (Meredith et al., 2011) also deactivated a visual area that normally mediates visual detection (posteromedial lateral suprasylvian visual area). This deactivation of the visual cortex resulted in complete abolishment of visual task performance in both deaf and hearing cats (Figure 6B – middle). This last finding provides conclusive evidence that auditory cortex cannot substitute for low-level visual cortex. Thus, a categorical switch of the processing capacity of a cortical territory from one sensory modality to another does not happen. Instead, a more subtle phenomenon seems to be in operation, whereby higher-order processing capacities that can operate across modalities must piggy-back on preserved modality-specific processing, in this case, visual cortex. This conclusion is similar to the one that Sur and colleagues came to at the end of their seminal paper (Sharma et al., 2000), where they posited more general cross-modality processing as an explanation for their results.

Consistent with the emphasis we are making on the differential potential of higher- versus lower-order cortex to aid another modality, the deactivation studies highlighted above (Lomber et al., 2010; Meredith et al., 2011) also tested the effect of A1 deactivation on the various visual tasks (Figure 6B – bottom). This did *not* result in significant performance change. The lack of functional impact of A1 deactivation is also consistent with other electrophysiological studies concluding that cross-modal activity is absent in A1 (Kral et al., 2003). This is despite the fact that the deprived A1 is more easily excitable (and less inhibited) (Butler and Lomber, 2013). Again, this result may not be so surprising once one sees these cross-modal effects as indicative of higher-order modality-invariant computations, given that primary A1 or V1 *are* much more modality specific.

Overall, this body of research provides a physiological explanation as to why functional cross-modal plasticity is restricted to higher-order areas. Due to the behavioural pressure induced by deafness, cognitive mechanisms triggered by task-related contextual processing (e.g. attention, executive control, reinforcement learning) are likely to trigger top-down compensatory control on areas that can computationally process residual inputs, such as those relating to vision. This upregulation will include higher-order auditory cortex as it has a general capacity to process visual input. Critical periods will likely support this process better. Notably, low-level cortex is not as influenced by frontal regulation (Yusuf et al., 2022; Yusuf et al., 2020), providing another explanation why effects in A1 will be minimal (Glick and Sharma, 2017). It appears that low-level sensory cortex is not pluripotent, even in the extreme case of congenital deprivation.

Another important advantage of the deaf cat model over the previous examples we have discussed so far is that hearing can be artificially restored, thanks to advances in the development of cochlear implants. This provides an exciting opportunity to determine whether cross-modal plasticity following congenital deprivation can switch over the functional affiliation of a given cortical area (from auditory to visual). In an electrophysiological study of the DZ (Figure 6A) – one of the higher-order auditory areas showing cross-modal plasticity in deaf cats, auditory responses to cochlear implant stimulation were compared between adult hearing and congenitally deaf white cats (Land et al., 2016). The neural responses to the restored auditory input were not different between deaf and hearing cats (Figure 6C). While a few neurons in the deaf cat continued to show visual responses, these were much fewer than the neurons responding to the new auditory input. This and similar studies provide strong demonstration that the auditory system develops functionally even in the absence of any hearing. This should come as no surprise, considering the enormous success of cochlear implants. Profoundly deaf children, even those with congenital hearing impairments, are able to acquire perception (e.g. speech comprehension) following cochlear implants (see Glennon et al., 2020, for review). While converging evidence demonstrate substantial benefit for implanting cochlear implants at infancy (<12 months) (Sharma et al., 2020), a reasonable amount of hearing can also be restored in older children (>7 years) (Gilley et al., 2008). As such, we see the body of literature relating to the deprived auditory cortex as strongly supporting the interpretation of plasticity (strengthening of existing circuitry), as opposed reorganisation (building of new connectional fingerprints).

### Use-dependent magnification of cortical finger representations in experts

So how do blind people (or deaf cats) outperform sighted individuals on a range of perceptual tasks, if it is not due to extra computational resources provided by the ‘reorganised’ cortex of the deprived modality? An alternative theory, which we wish to also disprove, is that this heightened perceptual acuity is due to practice-induced reorganisation in the primary sensory cortex (Birklein et al., 2015). That is to say, for example, that reorganisation in the blind occurs in the primary somatosensory cortex (S1) rather than V1.

S1 is organised topographically, with adjacent body parts (e.g. fingers) represented next to each other in cortex (Figure 8). The exact boundaries between fingers in the hand map are thought to be determined by Hebbian-like plasticity due to de/synchronisation of input. For example, electrophysiological studies in monkeys showed that suturing two fingers together (syndactyly; Clark et al., 1988; see also Allard et al., 1991) or repetitive co-stimulation across adjacent ﬁngers (Wang et al., 1995) blurs the boundaries between their cortical territories. Conversely, intensive tactile stimulation to one finger results in increased representation of the trained skin surface (cortical magnification), as demonstrated both by experimental training (Byl et al., 1997; Byl et al., 1996; Jenkins et al., 1990; Recanzone et al., 1992a; Recanzone et al., 1992b) and by natural exposure (Sterr et al., 1998; Xerri et al., 1994). Considering the importance of tactile information for blind individuals in everyday life (e.g. for Braille reading, obstacle avoidance using a cane, and object recognition), it is not surprising that researchers have hypothesised that they should show cortical magnification of their S1 hand representation relative to sighted individuals. This hypothesis calls for a demonstrable change in S1 finger map boundaries, that is, cortical remapping. We first consider the evidence for and against remapping in S1 of blind individuals, and we later consider the case of expert musicians.

**Figure 7.**
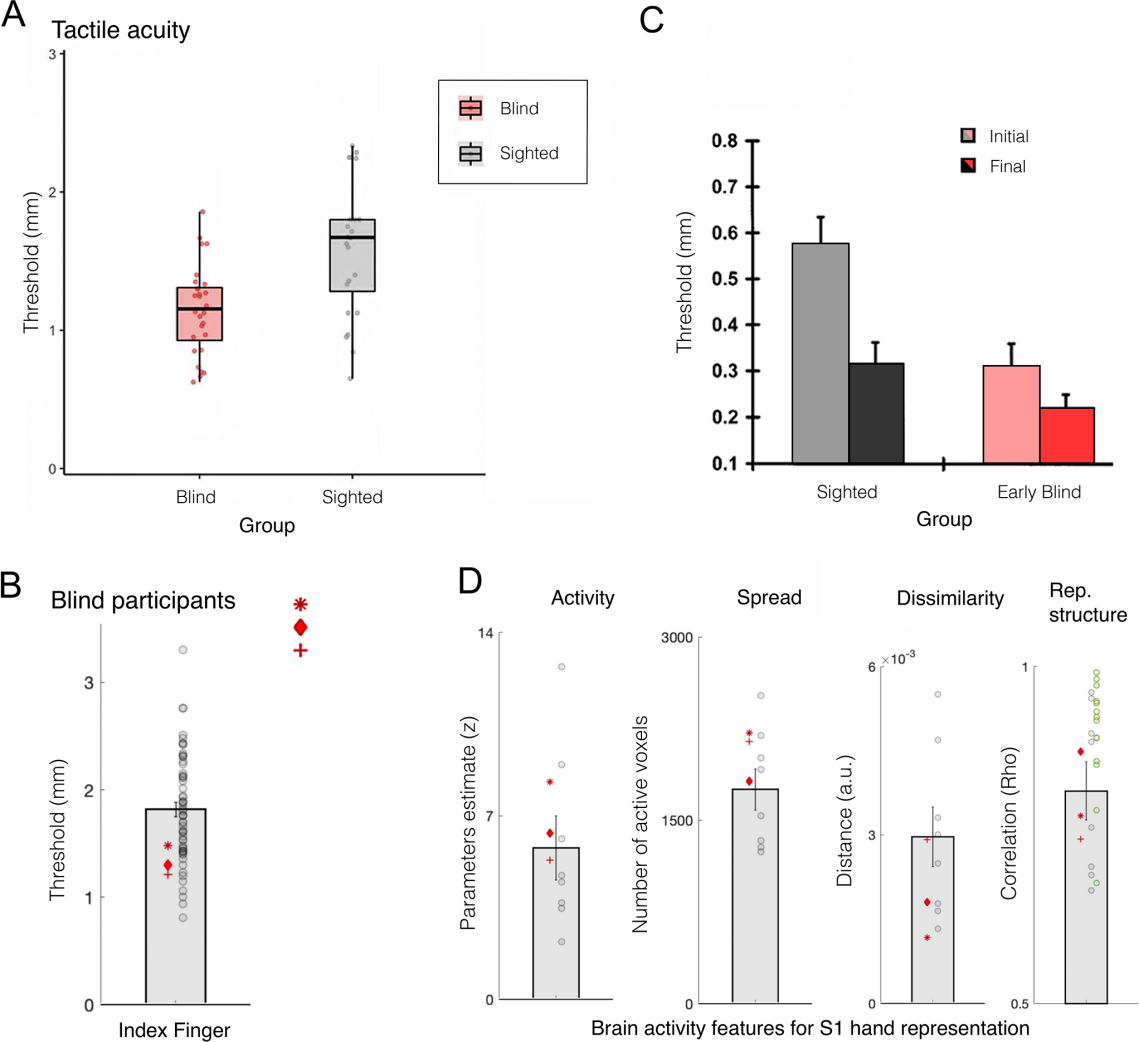
Finger acuity in blind individuals doesn’t relate to map changes to somatosensory cortex. (**A**) A replication of several classical studies showing that blind people (pink) exhibit on average better tactile acuity relative to sighted controls (grey), as measured using a tactile gratings discrimination task, where participants are asked to discriminate between two grating orientations applied on their index fingertip. This study finds that blind individuals exhibit lower tactile threshold, relative to sighted controls, indicating they have better sensory perception. (**B**) A replication study using similar methodology in three blind participants with bilateral anophthalmia (meaning their eyes never developed) against a large sample of sighted controls. Just by increasing the sample of the control participants, this study finds that, while acuity of the blind individuals (red markers) is significantly superior, their performance still falls within the normal range of sighted controls (grey circles). (**C**) A study examining the influence of training of tactile pattern discrimination on tactile acuity. In keeping with other research, significant group differences were found at the baseline session between the sighted and early blind groups. However, following several days of training on the task (up to 4 consecutive days), performance across groups leveled, and no significant group differences were found. This study highlights that practice could have contributed to the previously observed performance advantages in blind individuals (though we note that a statistical interaction between groups and sessions was not reported in this study). (**D**) A range of 7 T functional MRI (fMRI) measures for finger representation in primary somatosensory cortex (S1) for the anophthalmia blind individuals shown in (**B**). S1 finger representation was elicited by applying touch to each of the hand’s fingertips, and compared between the blind individuals and sighted controls in the following domains: activity – net activity level in the hand territory (averaged univariate parameter estimate for all fingers versus rest); spread – number of active voxel (surviving correction for multiple comparisons) in the hand territory; dissimilarity – cross validated Mahalanobis inter-finger distances using representational similarity analysis; representational structure – derived from the distances across all finger pairs, and correlated with a template for canonical hand representation. The green circles show the representational structure of control participants scanned using a slightly adjusted protocol. Across all measures, the S1 hand representation of the blind individuals is comparable to the sighted controls. Rep.=representational. (A) is reproduced from Figure 3 from Radziun et al., 2023; (B) is reproduced from Figure 1 from Wesselink et al., 2021; (C) is reprinted from Figure 2 from Grant et al., 2000; (D) is adapted from Figure 2 from Wesselink et al., 2021.

Blind individuals’ fingers, in particular the reading fingers of Braille readers, have been reported to have increased representation in S1, in terms of spatial spread of activity (Pascual-Leone et al., 1993a; Pascual-Leone and Torres, 1993b), strength of activity (Giriyappa et al., 2009), or shifted inter-finger balance (Sterr et al., 1998). This has been interpreted as a positive adaptation towards more effective tactile processing for people who rely more on touch in their daily lives. But the evidence in support of this simple idea is not convincing. For example, Gizewski et al., 2003, found little difference in how primary sensorimotor cortex is activated during finger tapping in blind and sighted individuals. Another study showed no statistical differences in average S1 activity for early blind and sighted individuals during vibrotactile stimulation; though their data may show a weak effect of blindness on the spatial spread of activity this was not statistically tested (Burton et al., 2004). Perhaps the most detailed investigation of S1 finger representation to date was reported by Wesselink and colleagues, using three individuals who were born without eyes (Wesselink et al., 2021; Figure 7B and D). Here, they examined multiple features of the hand map, using high spatial resolution (7 T fMRI), as well as multivariate outcome measures to probe the information content in the hand territory. They found that, despite clearly superior tactile acuity, the three individuals did not show more pronounced finger maps than sighted controls in any of the tested measures. Another recent study, testing for hand representation in two macaques who were near blind in the first year of life, also confirms that their hand representation was not different from control animals (Arcaro et al., 2019).

Therefore, the evidence that blind individuals’ heightened sensory abilities are anchored in cortical map changes, either via cross-modal plasticity (as reviewed in section ‘Cross-modal plasticity in congenitally blind individuals’ above; see also Singh et al., 2018) or unimodal plasticity (as reviewed here) is largely anecdotal and requires reconsideration. Instead, growing research shows that the superior perceptual performance often seen in blind individuals, for example, in tactile acuity (Grant et al., 2000; Figure 7A-C) or echolocation (Dodsworth et al., 2020) could be due to training effects – in their daily lives, blind individual will dedicate more time and attention to leaning to extract more out of these sensory cues, making them better at these perceptual tasks in the lab. But if sighted individuals are trained to improve their performance on these tasks, they end up performing just as well as blind individuals, as shown in Figure 7C. In other words, it doesn’t seem that blind people have any advantage over sighted individual when it comes to the effects of practice. Indeed, as visualised in Figure 7B - the a priori superior performance shown by the blind participants did not fall outside the range of the sighted population.

Assuming the increased abilities identified in blind individuals are due to perceptual learning, we can still ask whether perceptual learning provides a potential mechanism for triggering cortical remapping – could perceptual learning lead to change in the boundaries of sensory maps? Foundational work has indicated that perceptual learning is constrained to the specific trained stimulus features, such as a given spatial location or frequency. As these training features often reflect the organisation features of their underlying cortical sensory maps, it has long been assumed that perceptual learning relies on local processing in sensory cortex (Sagi, 2011). Therefore, training to improve perceptual abilities, such as in the case of blind individuals, was assumed to rely on local tuning of the receptive field properties in the sensory maps. However, the empirical evidence to support this process is much more limited. For example, one of the first studies documenting electrophysiological correlations in V1 of monkeys for orientation identification did not find a shift in preferred orientation, which would be required to change V1 orientation map boundaries (Schoups et al., 2001). Instead, the authors identified a much more subtle change in the slope of orientation tuning curve in a subset of neurons, which was estimated to explain less than 10% of the behavioural perceptual changes. Later work has demonstrated that perceptual gains reflect the improved ability of higher-level decision-making areas to read out task-relevant sensory information (Dosher and Lu, 1998; Law and Gold, 2008). More recent thinking conceptualises perceptual learning as a multi-stage process, highlighting the contributions of multiple brain areas beyond low-level sensory cortex (for review see Maniglia and Seitz, 2018). Therefore, while perceptual learning is likely supported by some local plasticity in primary sensory cortex, these changes are subtle, and therefore are unlikely to cause map changes at the macro scale.

What about other experts that are capable of exceptional skills due to extensive practice beginning early in life? Performing music at a professional level is an example of this. It has been suggested that training to play a musical instrument from an early age will cause reorganisation of finger maps. For example, string musicians were shown to produce a stronger current dipole (the net effect of ionic current) when their left little finger (D5) was stimulated with light pressure (Elbert et al., 1995) (see Pantev et al., 1998, for similar findings in pianists’ auditory cortex). Importantly, the strength of this dipole was substantially correlated (r=0.79) with the age at which participants started training to play their instrument – the earlier training started in childhood, the stronger activity was found (some readers might find the following reference useful to contextualise this striking correlation; Vul et al., 2009). This, and related findings, helped promote the idea that plasticity can be more readily harnessed to support the acquisition of new skills at earlier stages of development.

However, such interpretation requires a conceptual leap of faith. First, it is just as likely that people with greater capacity for sensory processing will persist in their musical practice for longer, thus reversing the inferred causality without the need to invoke plasticity. Without longitudinal studies, these two possibilities cannot be teased apart. Second, the age of onset of musical training is generally confounded by the degree of expertise: early starters usually have been practicing for a larger amount of time than late starters and thus, on average, should have a greater level of expertise. As such, we don’t think there’s sufficient evidence to support the idea that early-life practice produces (or is facilitated by) greater capacity for brain plasticity in somatosensory cortex than later in life. Moreover, recent research using RSA looking at inter-finger representation found that the S1 and primary motor cortex (M1) finger representation in professional musicians is normal, even when considering those with focal dystonia (Sadnicka et al., 2023), a condition which was previously widely considered to be due to maladaptive brain reorganisation from over-practice (Elbert et al., 1998). Other research comparing amateur musicians to non-musicians also found no significant differences in S1 (Ogawa et al., 2019). So, despite much research, there is no cumulative evidence of map expansion in hand somatosensory cortex due to early life skill training. And even if this evidence had been shown, there is no need to either assume its causal relevance to the skill or to invoke reorganisation.

### Finger remapping in adult monkey somatosensory cortex

**Figure 8.**
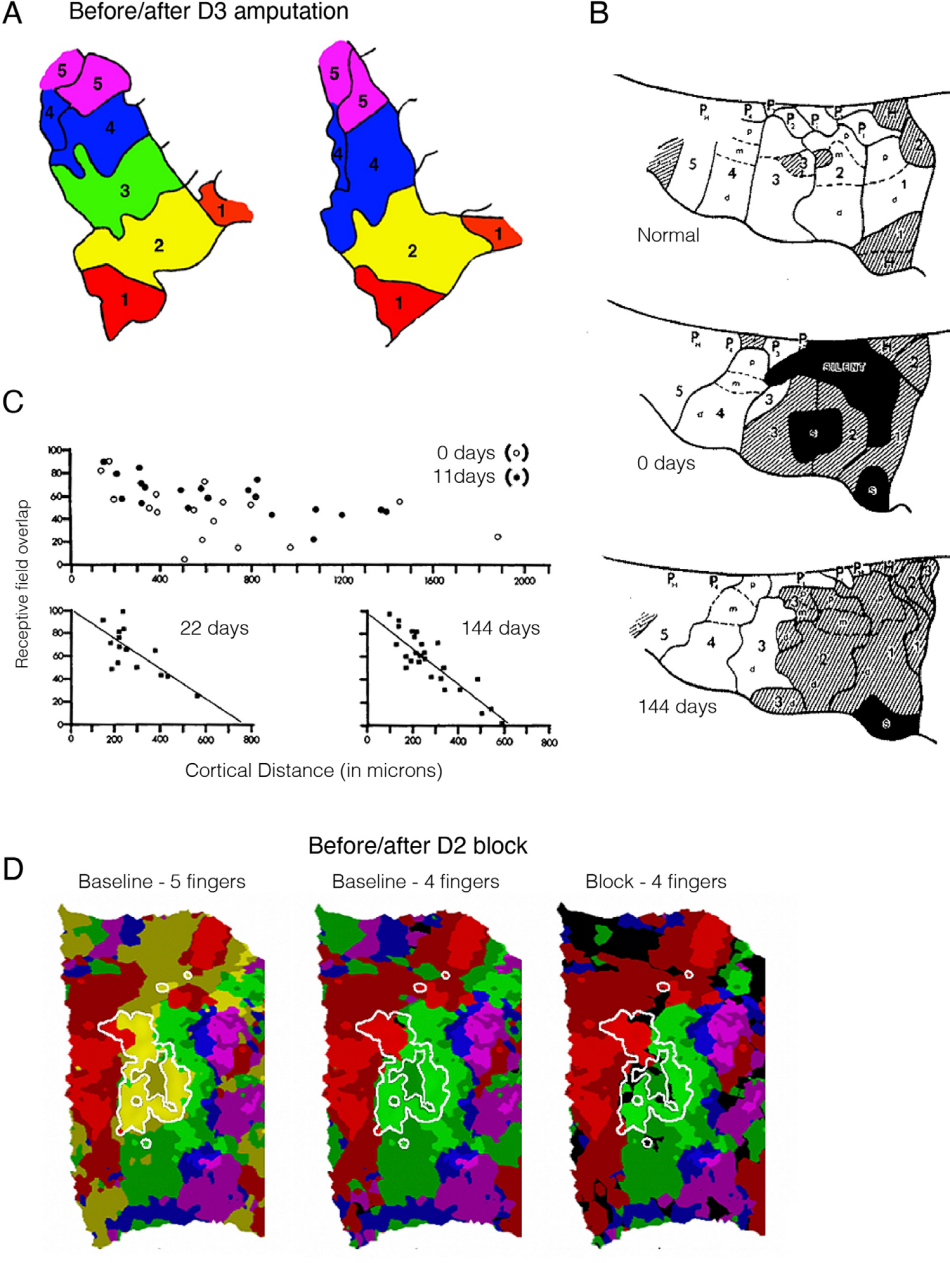
Remapping in the primary somatosensory cortex (**S1**) hand map does not imply reorganisation. (**A**) A finger map in the monkey’s S1, created using a winner-takes-all single-cell recording approach. The map shows clear boundaries between sub-regions dedicated to processing inputs for each of the five monkey’s digits (**D1–D5**), with neighbouring digits on the hand next to each other in the cortical map. A classical electrophysiological result from Merzenich and colleagues, showing that following digit amputation (**D3**), the deprived (green) territory gets ‘taken over’ by its cortical neighbours (D2 – yellow and D4 – blue). (**B**) Remapping following median nerve transection, immediately and (close to) 5 months following deprivation. Top: Typical finger map in the monkey’s S1 hand territory. Each of the five digits (**D1–D5**) is segmented into its three pads (proximal, medial, dorsal), and its related pad on the palm of the hand. Cortical territory of the glabrous skin (the palm of the hand) is indicated in white, whereas hairy skin (back of the hand) is hatched. Normally, the median nerve mediates sensory information from the glabrous skin of digits D1-D3. 144 days following transection, the cortical territory for the median nerve was activated by touching hairy skin, however most of these activation changes were already noticeable immediately following the nerve transection (day 0), indicating the hairy skin input was already present in the median nerve territory. (**C**) Retuning of D4 receptive fields in the ‘remapped’ cortex following median nerve transection. In the first few weeks following transection, the receptive field sizes were broadly overlapping across cells, irrespective of their cortical distant from each other. Within a few weeks, the topographic features were restored. Therefore, remapping here can be described as the process of tuning and refining pre-existing receptive fields which already have the innate capacity to process the ‘remapped’ input. (**D**) Remapping can simply be the consequence of methodological choices. Winner-takes-all assignment of voxels to fingers (based on 7T functional MRI [fMRI] activity), in the S1 cortical hand territory of an example participant (colours as in (A)) before (left - baseline) and after (right - block) pharmacological deafferentation of D2 using a nerve block. The white outline shows a highly selective D2 cluster, as identified independently. When comparing four fingers (i.e. excluding D2, following the nerve block) instead of all five, we see ‘invasion’ of the neighbouring fingers (D1, red; D3, green) into the D2 territory (white outline), as shown on the right. Crucially, further analysis reveals that this is due to the statistical procedure, rather than the D2 nerve block: performing the same analysis (comparing four fingers and excluding D1) on the baseline dataset resulted in similar ‘remapping’ as found in the block map, as shown in the middle panel. Bright and shaded colours indicate activated (Z>0) voxels inside and outside the independently localised finger clusters, respectively; black indicates below-zero activity. Images were adapted from: (A) is reprinted from Figure 1 from Merzenich et al., 1984; (B) is reprinted from Figure 7 from Merzenich et al., 1983a, and (C) from Figure 13; (D) is reproduced from Figure 2 from Wesselink et al., 2022.

A famous claim for reorganisation comes from classic studies in adult monkeys showing changes in cortical finger maps following peripheral input loss. As shown in Figure 8A, following amputation of one finger (middle finger, D3, in the example below), the cortical neighbours of D3 – index (D2) and ring fingers (D4) were said to ‘take over’ the deprived D3 territory (Merzenich et al., 1984). Similarly, following median nerve transection (innervating the palm of D1-D3), the deprived territory became activated by inputs to the back of the hand (Merzenich et al., 1983a). A seminal study by Merzenich and colleagues provides us with one of the few glimpses into how this remapping process unfolds over time (Merzenich et al., 1983b). The ‘invading’ inputs into the deprived cortex happened instantaneously (Figure 8B), but the receptive fields of the displaced inputs underwent changes over the course of months (Figure 8C). Initially, the receptive fields of the displaced input were large and overlapping, and generally disorganised. But over weeks and months the receptive fields became more tuned, with linear scaling between cortical distance and the receptive field overlap between two neurons, resulting in a typical organisation of the displaced input (as also shown in Pons et al., 1991). It has been suggested that the initial stage of remapping is mostly powered by unmasking of silent inputs, due to rapid disinhibition of homeostatic plasticity mechanisms (Gainey and Feldman, 2017), while the slower process of receptive fields tuning is aided by Hebbian learning (Churchill et al., 1998). Evidence for similar remapping following finger amputation was also reported in humans (Oelschläger et al., 2014). That this remapping was demonstrated in the adult brain, well outside any critical period, sparked much interest and speculation about the brain’s capacity for reorganisation. Is this enthusiasm warranted?

Two important points to be considered are that most ‘remapping’ occurs immediately following the nerve transection (Figure 8B), and that different patterns of deafferentation lead to different remapping outcomes (Garraghty et al., 1994). Both points indicate that unmasking of existing connections is a key mechanism underlying the remapping phenomenon. Considered in this way, it is perhaps hard to understand why such expression of latent input should have ever been taken as evidence for reorganisation. Maybe this is because displaced input in the adult brain has been thought to be suppressed, and as such was considered functionally ‘ineffective’ (O’Leary et al., 1994). Indeed, it is worth asking - are these processes of unmasking functionally meaningful with behavioural consequences?

It has been hypothesised that by gaining a new cortical territory, the ‘invading’ skin surface (e.g. D2 and D4 in the example shown in Figure 8A) has access to increased computational resources which should enable heightened perception. This idea is akin to the concept of cross-modal plasticity, but arguably stands on more solid ground. This is because, unlike the architectural constraints restricting cross-modal plasticity, as discussed in sections ‘Experimentally induced rewiring of retinal input in newborn ferrets’ and ‘Cross-modal plasticity in congenitally blind individuals’, the organisational properties of somatosensory cortex are relatively homogenous across the hand map. Nevertheless, behavioural studies in humans testing such hypothesised perceptual gains have failed. For example, finger amputation does not result in lower detection thresholds or improved spatial acuity in the neighbouring fingers (Oelschläger et al., 2014; Vega-Bermudez and Johnson, 2002). Earlier reports for increased tactile acuity on the stump of amputees (e.g. Haber, 1958) have been subsequently challenged. Instead, evidence is emerging to suggest that potential perceptual advantages following deprivation might be driven by compensatory behaviour (Dempsey-Jones et al., 2019; Oelschläger et al., 2014), similar to the training gains we discussed in blind individuals in section ‘Use-dependent magnification of cortical finger representations in experts’. Thus, it could be that the observed change in activity due to unmasking is not sufficient for impacting behaviour. For example, a more recent study looking at remapping in deaf cats demonstrated that local territorial expansion does not impact downstream function or drive connectional reorganisation (Meredith et al., 2017). Instead, the local activity increase that is thought to drive remapping could simply reflect gain modulation. Another possibility we would like to entertain is that the reported changes to the boundaries of the maps are artefacts constructed by researchers.

As shown in the left side of Figure 8D, finger maps can be identified in humans with ultrahigh field (7T) fMRI . This simple winner-takes-all procedure derives an orderly map of the five fingers. The white boundary in the figure shows a subset of voxels showing the strongest selectivity for D2 over the other hand fingers, based on an independent localiser. In the right panel, the same procedure is repeated following peripheral nerve block of D2. Exactly as described in the electrophysiological studies, it is found that the previous territory of D2 (white boundaries) is now ‘taken over’ by the cortical neighbours – D1 (red) and D3 (green). But beyond the nerve block manipulation that has been used to produce the right panel, another stark difference between the left and right maps is the number of fingers that were competing in the winner-takes-all analysis. True to the mapping procedure used in previous studies, the winner-takes-all approach in the right is limited to the four unblocked fingers, that is, excluding D2. To even the playing field, the winner-takes-all analysis was repeated on the baseline dataset, while excluding D2. As shown in the middle panel, this resulted in a statistically identical map to the one on the right – found following the D2 nerve block. But, of course, at baseline, remapping did not happen. The reason the cortical territory of D2 seems to be ‘taken over’ by the neighbour is due to the fact that the information about these fingers was already there, but the way we define and draw the maps makes it seem as if it were not. In fact, further analysis in this study revealed that, at least over that limited timescale, there was no increased representation of the neighbouring fingers within the D2 area (Wesselink et al., 2022). So, as in the criticisms we’ve raised in sections ‘Recovery of language abilities after perinatal brain damage’ and ‘Monocular deprivation in kittens’, we have to question if the reported changes to map boundaries reflect a true physiological change. If not, then remapping might be an artefact of analysis, rather than a true biological phenomenon.

To be clear, we are not arguing that all evidence for remapping is artefactual. Instead, we propose that all-or-nothing changes to the boundaries of the map may be. The remapping of finger representation in the S1 hand map is likely supported by representational overlap across multiple fingers – underneath the apparent selectivity, single-finger representation in S1 is clearly more distributed than might be assumed from topographical mapping studies (Thakur et al., 2012; Tommerdahl et al., 2010). Accordingly, if the neurons at the inter-finger borders of the hand map were already natively broadly tuned to receive inputs from the neighbouring finger, as suggested by human fMRI studies (Schellekens et al., 2021; Wang et al., 2021), then the observed changes to the finger boundaries do not indicate reorganisation. Instead, the modulated receptive fields could be achieved by adjustment of the parameters that are already shaping the neural response. Such synaptic reweighting can be contained within a standard account of neural plasticity without having to invoke reorganisation.

### Large-scale remapping of somatosensory cortex in amputees

**Figure 9.**
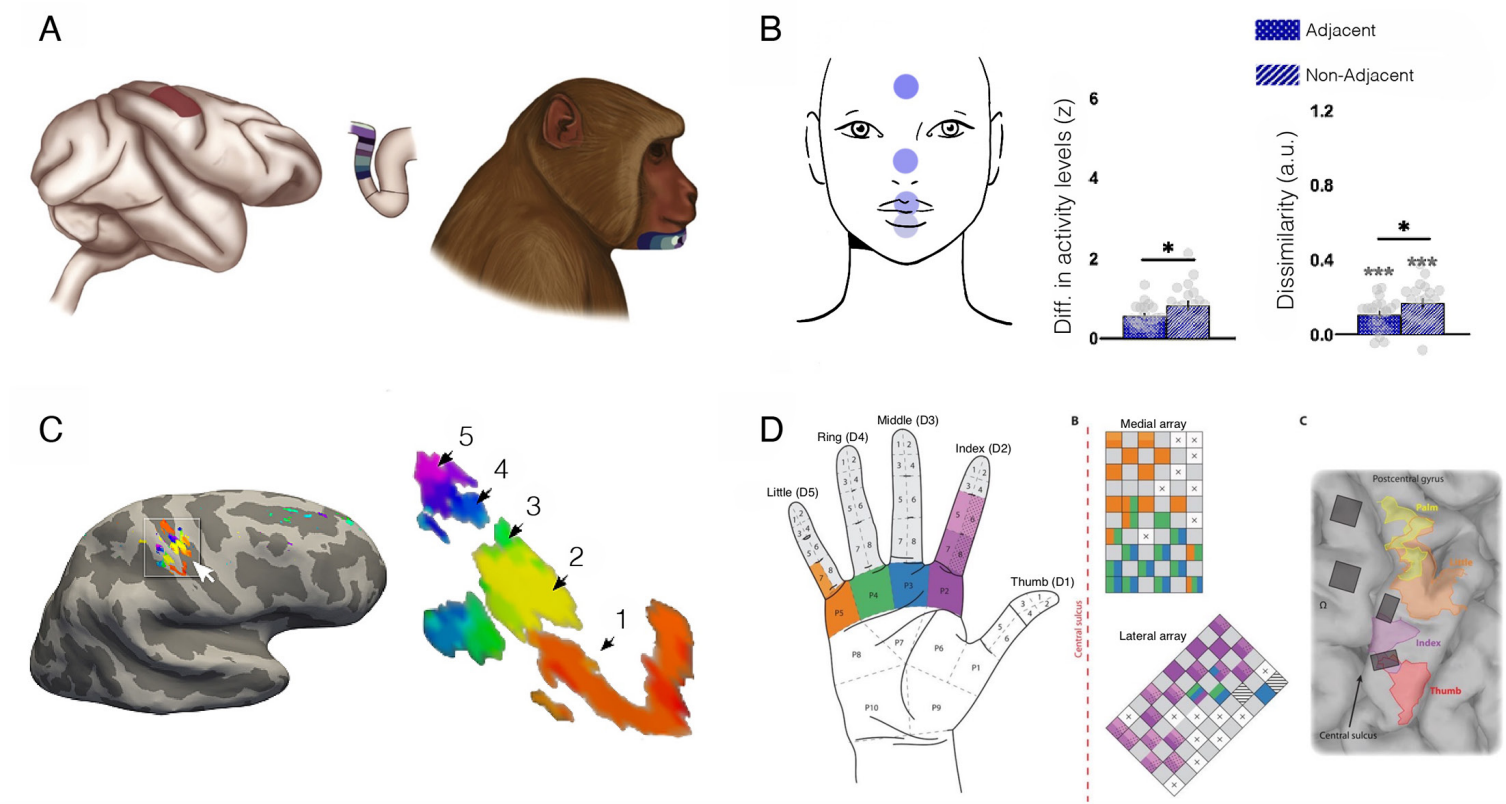
Face and hand representations can remain stable following arm deafferentation. (**A**) The primary somatosensory (S1) hand area, shown from a lateral view (left) and through a cross-section of the central sulcus (middle). The colours along the postcentral bank of the sulcus reflect receptive fields that were mapped to the lower face of a monkey (right) with an arm deafferentation in the classical electrophysiological finding by Pons et al., 1991. The study showed topographic remapping of the monkey’s chin in the deprived S1 hand territory following arm deafferentation. (**B**) Topographic features for face representation in the cortical S1 hand territory of intact (non-disabled) human adults. A key feature of topographic mapping is that non-neighbouring representations are more distinct, providing an opportunity to examine whether topographic motifs can be identified for the face even in the typical hand area. Using fMRI and facial movements, univariate and multivariate activity was compared across four facial parts (forehead, nose, lips, and tongue; highlighted on the top left). Greater activity differences (left) and dissimilarity (cross-validated Mahalanobis distances using representational similarly analysis, right) between facial parts that are non-neighbouring (e.g. forehead and lips) were confirmed in the S1 hand territory, relative to neighbouring representations (e.g. forehead and nose). This indicates that topographical features of the face are already present in the hand territory prior to deafferentation. (**C**) S1 finger map of the missing (phantom) hand in an upper limb (above elbow) amputee who lost his hand over 30 years before, as measured by 7 T fMRI (travelling wave analysis). The amputee was asked to move each of his phantom fingers, and the resulting analysis identified clusters showing clear selectivity for each of his five fingers in the central sulcus and postcentral gyrus around the hand knob. The numbers correspond to clusters showing selectivity to each of the five digits (1=D1, 5=D5; colour code as in Figure 8). This finding indicates that hand representation persists following amputation. (**D**) Projected field maps for the hand of a tetraplegic patient in response to intracortical microstimulation to S1. The left panel shows the hand regions in which evoked sensations were reported. The middle panel displays the two electrode arrays that were implanted in S1. The colour code corresponds to the parts of the hand where sensations were elicited. The right panel shows the electrode implantation in the cortex. Microstimulation in the deprived S1 territory evoked a topographic sensation pattern, providing causal evidence that S1 functionally represents the hand, despite deafferentation. Figures were taken from: (A) is reproduced from Figure 1 from Makin and Bensmaia, 2017; (B) is adapted from Figure 3 from Muret and Makin, 2021; (C) is reproduced from Figure 1 from Kikkert et al., 2016; (D) is reproduced from Figure 1 from Flesher et al., 2016.

It is interesting to ask what will happen to the receptive field of a finger neuron if input from the entire hand and arm is lost due to arm deafferentation or amputation. Multiple studies in adult monkeys following deafferentation, amputation, and spinal cord transection have shown that neurons in the hand territory become activated by inputs from the lips and jaw (Florence and Kaas, 1995; Jain et al., 2008; Kambi et al., 2014; Pons et al., 1991). Normally, information from the hand and the face is conveyed to S1 via two separate brainstem pathways – the cuneate and the trigeminal nerve, respectively. The hand and face cortical territories are in fact separated by a dividing wall of tissue called septa, which creates a myelin border, meaning very few axons project across the face-hand boundary in intact animals (Chand and Jain, 2015). It is this presumably strict separation of input to the hand and face cortical territories that had led to the interpretation of remapping of facial input into the deprived hand territory as strong evidence for reorganisation in the adult brain. Moreover, the newly ‘remapped’ lower face representation in the deprived hand territory has been shown to be topographically organised. Considering the stark representational differences between the hand and face, this evidence appears to meet one of the strict criteria we set out for reorganisation and haven’t encountered so far – a region responding to a qualitatively novel input (Figure 9A).

How does the face input reach the hand region? This presumptive cortical reorganisation was originally thought to be the result of widespread sprouting of new intracortical connections, which could hypothetically provide new input to the deprived hand cortex (Florence et al., 1998; Pons et al., 1991). However, later evidence pointed to subcortical structures, and the brainstem in particular, as the driver of the displaced input (Jones and Pons, 1998). Specifically, face activation in the former hand territory is abolished when the cuneate nucleus is inactivated, but not when the S1 face territory is inactivate (Kambi et al., 2014). Indeed, deafferentation does not cause any measurable increase in cortical boundary-crossing axonal projections (Chand and Jain, 2015). In other words, the hand territory of the cortex receives input from the cuneate (via thalamus) just as before the amputation, but this pathway is now also activated by trigeminal input. The activation of the hand territory when the face is touched can therefore be explained without any need to invoke cortical reorganisation. Even the topographic organisational feature of the face documented within the deafferented monkeys’ hand territory has been seen in the S1 hand region of neurotypical human adults (Muret et al., 2022). The takeaway message again is that, if topographic face information is already present at baseline (as shown in Figure 9B), and if amputation results in gain modulation of facial activity via brainstem circuits, then the canonical findings in the monkeys shown in Figure 9A do not entail establishment of a novel input.

It is also important to note that the remapped lip activity in monkeys is substantially weaker than that observed within the native facial cortical territory (Kambi et al., 2014), and that research in human arm amputees has not identified compelling evidence for facial activity in the missing hand cortex (Kikkert et al., 2018; Makin et al., 2013; Root et al., 2022; Valyear et al., 2020). Nevertheless, the presumed remapping of the face into the hand territory has been theorised to trigger a curious phenomenon where some amputees report feeling referred touch sensations on the missing hand, following touch to the lower part of the face (Ramachandran, 1993; Ramachandran et al., 1992). This interpretation received tremendous attention both from the scientific community and the general public (Ramachandran and Blakeslee, 2005). But the findings should be interpreted with caution. Specifically, in these and related studies, the participant is asked by the experimenter to describe sensations on their phantom hands, with no consideration of suggestibility effects or response compliance, which are bound to affect the participant’s reports. Indeed, when the experimental designs were extended to include control stimulation sites (across the entire body; Grüsser et al., 2004; Knecht et al., 1996), control suggestions (that referred sensations could also be elicited on the intact hand; Amoruso et al., 2023), or using a control group (intact controls or even individuals who were born without a hand; Amoruso et al., 2023), the referred sensation phenomenon was no longer uniquely related to the amputation. Indeed, under carefully controlled experimental settings, amputees do not report more frequent referred sensations on their phantom hand relative to either controls or indeed their intact hand. Finally, there is no evidence that activation in the deprived hand territory in response to facial input has any functional consequences (see Makin, 2021, for a critical discussion on remapping and phantom limb pain). Therefore, this example doesn’t meet any of the criteria we set out in our definition of reorganisation, relating to input, novel computation, or functional read-out.

Given all this refutational evidence, why has the neuroscience community spent so much time and effort to study facial representations in the missing hand territory of amputees? A key problem that is shared across sensory deprivation studies is that once the relevant input is lost, for example, due to hand amputation, it becomes difficult to investigate this region directly. Therefore, scientists resort to studying the deprived cortex indirectly, by examining how it responds to input from spared (intact) body parts. But such a trial-and-error approach can lead to spurious findings. One solution to this problem is to study surgical procedures, such as hand transplantation, that restore the missing hand input even years after amputation. Human studies following hand restoration have suggested that the somatotopic representation of the restored hand remains intact. For example, tactile stimulation of an amputee’s transplanted arm elicited activity within the (previously deprived) hand territory comparable to that observed in two-handed controls, with no evidence for an expanded face representation (Frey et al., 2008) (see also Giraux et al., 2001, for equivalent results in motor cortex). Similarly, following targeted muscle reinnervation of a severed nerve, amputees showed the representational motifs of the canonical finger map (Serino et al., 2017) (see also Liao et al., 2016, for related evidence in monkeys). These findings were originally interpreted as further evidence for the dynamic abilities of the adult S1 to dramatically reverse its presumed reorganisation based on changing input. However, we believe this result is better interpreted as evidence for the long-term persistence of the original cortical map (Makin and Bensmaia, 2017).

As an alternative framework to reorganisation, cumulative evidence suggests that even when deprived of its normal input by amputation, the canonical representational structure and function of sensory cortex remains intact. Adult amputees report lingering phantom sensations of their missing limb (Weeks et al., 2010) and phantom hand movements (or at least attempted movements) selectively activate deprived sensorimotor cortex (Makin et al., 2013; Raffin et al., 2012). Studies with high-resolution (7T) fMRI revealed persistent finger maps in S1 for the missing hand even decades after amputation – as demonstrated in Figure 9C (Kikkert et al., 2016). Multivoxel analysis techniques revealed that the unique configuration of inter-finger representational structure (Ejaz et al., 2015) is entirely preserved following amputation, independent of time since amputation (Wesselink et al., 2019). In fact, the typical hand representational structure was also found in the few amputees who did not experience any phantom sensations, suggesting an invariant cortical organisation rather than a second-order effect of aberrant sensory input. Phantom hand activity was successfully used to decode a range of hand gestures performed with the phantom hand, suggesting a preserved rich representational content of the missing hand in the deprived cortex (Bruurmijn et al., 2017). But perhaps the strongest support for preserved hand representation despite sensory deprivation is provided by assessing the sensations elicited by direct stimulation to the cortex in deafferented patients (Gunduz et al., 2020), or to the peripheral nerves in amputees (Bensmaia et al., 2023). Intracortical microstimulation within the S1 hand territory of a tetraplegic patient elicited tactile sensations localised to the insensate hand (as shown in Figure 9D), and not to any other body part (Flesher et al., 2019; Flesher et al., 2016). Together with the high incidence of phantom sensations, these studies illustrate a high level of functional preservation across the sensorimotor pathway, with stable processing both at the representational and phenomenological levels. Thus, even the seminal example of phantom sensations used to argue for cortical reorganisation does not bear up to scrutiny.

### Changes related to recovery from ischemic stroke in primary motor cortex

**Figure 10.**
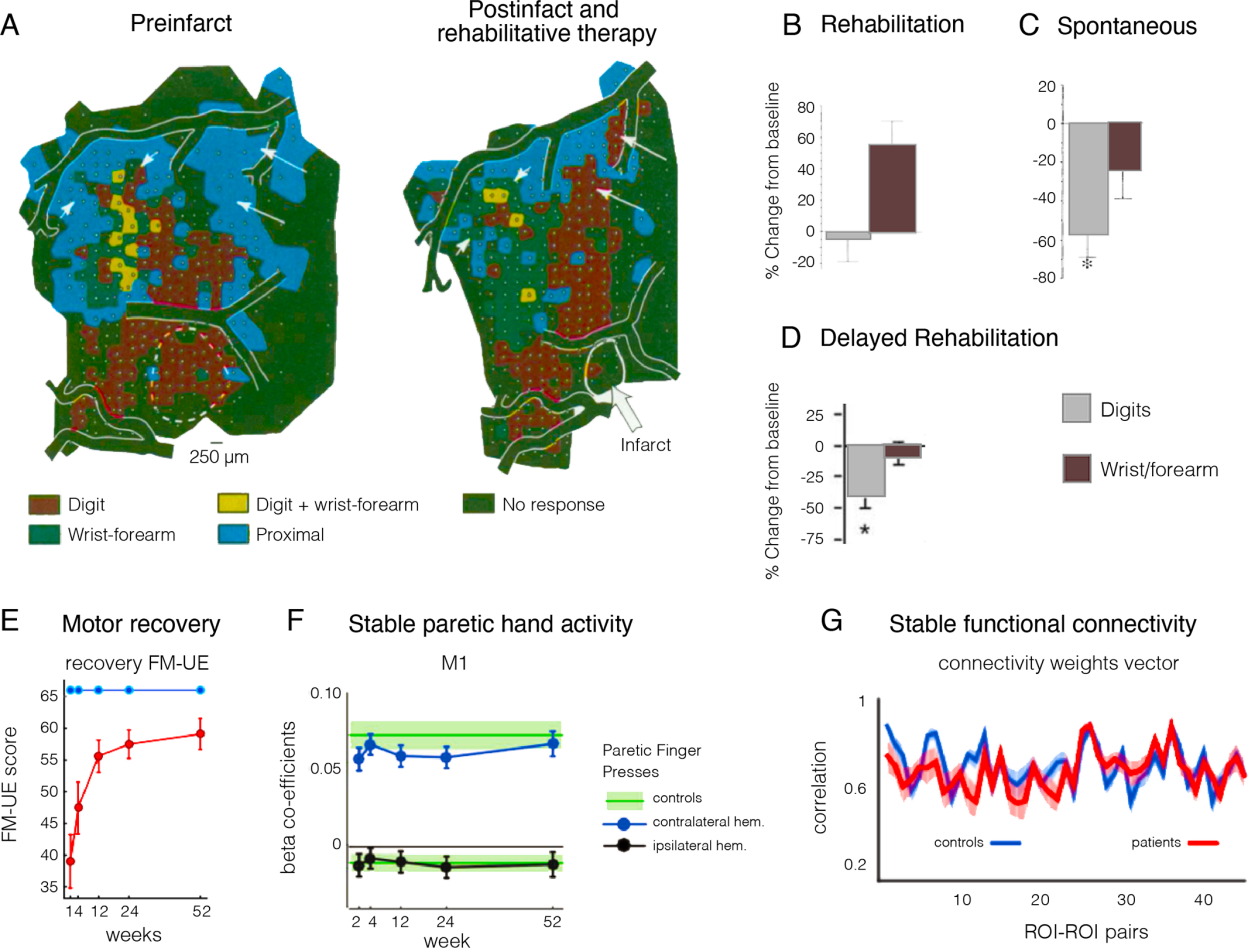
Remapping of hand and arm representation following focal lesion doesn’t reflect motor recovery. (**A**) Upper limb map in a monkey showing typical hand representation prior to M1 lesion (shown by white dashed line) and remapping of the hand representation after lesion to the digit area and training-based recovery. (**B–D**) Changes in the percentage of cortical territory dedicated to digit (dark) and writs/forearm (light) representation following M1 lesions to the digit territory, coupled with (**B**) training-intensive rehabilitation therapy, (**C**) spontaneous recovery, and (**D**) delayed training. In all three studies, motor behaviour was restored to comparable levels, yet the underlying map changes were radically different, suggesting that map changes are not straightforward correlates of motor recovery. (**E–G**) Functional MRI (fMRI) longitudinal studies, through the acute (1–12 weeks) and into the chronic stage (24–52 weeks) of stroke patients. (**E**) Recovery on the Fugl-Meyer upper extremity (FM-UE) scale shows substantial recovery of hand function by week 52 (red line). (**F**) Evoked BOLD activities in contralateral and ipsilateral M1 cortex induced by single-finger key-presses by the paretic hand. (**G**) Resting-state functional connectivity pairwise profile across five regions of interest (ROIs) (S1, M1, premotor dorsal and ventral, and supplementary motor area) for the left and right hemisphere for patients (red) and controls (blue). No systematic differences in connectivity patterns of patients and controls were found in the early subacute recovery period. Put together, despite significant motor recovery over the course of the acute phase, the activity and connectivity profiles of the paretic hand in the M1 hand area were stable over time. Modified from: (A) is adapted from Figure 2 and (B) is adapted from Figure 3 from Nudo et al., 1996b; (C) is adapted from Figure 3 from Nudo and Milliken, 1996; (D) is adapted from Figure 3 from Barbay et al., 2006; (E) is adapted from Figure 2 from Branscheidt et al., 2022; (F) is reproduced from Figure 3 from Ejaz et al., 2018; (G) is reproduced from Figure 3 from Branscheidt et al., 2022.

Another notion that we wish to demystify is that the significant degree of motor recovery that can occur after stroke, and other forms of brain injury, is attributed to cortical reorganisation in motor areas. This assumption is directly analogous to what is presumed to occur in sensory areas. In a series of seminal studies by Nudo and colleagues, adult squirrel monkeys were pre-trained on a hand dexterity task and then small sub-total M1 ischemic lesions (infarcts) were made within the M1 hand representation. In a now classic study, intense dexterity training was resumed 5 days after the cortical infarct and continued until the monkeys regained their former dexterity, which occurred at around 3–4 weeks post-stroke. The authors used intracortical microstimulation to derive detailed maps of the hand representation of three monkeys before the infarct and after motor recovery was achieved. Unlike the sensory maps discussed so far in monkeys, where the measured activity might not be directly relevant to that area’s function, stimulation is arguably more causally relevant as it shows that the cortical territory can drive movement in the corresponding effector. This important distinction, relative to the sensory remapping we discussed so far, will in principle fulfil our third criterion set out for ‘true’ reorganisation – that the changed local processing is read out coherently to guide novel behaviour. Indeed, when compared with the pre-infarct map, digit and wrist movements were elicitable by stimulation of adjacent regions that formerly only elicited elbow and shoulder movements (Nudo and Milliken, 1996; Figure 10A–B). These mapping results have been taken as strong proof of training-induced cortical reorganisation.

However, the causal importance of motor map expansion to recovery is called into question by a previous study by the same authors, in which monkeys also reached pre-operative levels of dexterity but without remapping. In this study, training was not provided, instead recovery was spontaneous. Although recovery understandably took longer – about 2 months to return to pre-operative levels of hand dexterity (Nudo et al., 1996a), in this case there was widespread *reduction* in the areal extent of digit representations adjacent to the stroke, with their apparent replacement by elbow and shoulder representations (Figure 10C). Thus, recovery is not dependent on map expansion and hence reorganisation does not need to be invoked to explain it. This is reminiscent of studies in animal models of motor skill learning, which have also shown that training leads to map expansion in motor cortical territories (Kleim et al., 1998; Nudo and Milliken, 1996) but the persistence of this expansion is not necessary for retention of the skill. Two studies showed that maps expand transiently but then contract whilst improved skill is retained (Molina-Luna et al., 2008; Tennant et al., 2012). Overall, map changes are, at best, markers that training has occurred but are not the causal factor for skilled normal behaviour, let alone recovered behaviour.

A critical but perhaps subtle point is that the epiphenomenal nature of map expansions and contractions calls the entire notion of reorganisation into question. This is because expansions in fact indicate only a *quantitative* change in an already existing capacity, whereas one of the key tenets of the idea of reorganisation is that one should see a qualitative switch in the identity of a cortical neuron or area precisely *because* it is the cause of the recovered behaviour. That is to say, reorganisation is the phenomenon, and remapping is proof of the phenomenon. The former evaporates when the latter is invalidated.

Perhaps the strongest evidence against reorganisation for recovered behaviour comes from double lesion studies. Here, the residual primary motor cortex, which presumably should be recruited to support motor recovery, is lesioned or inactivated without a behavioural consequence. For example, two monkeys with total lesions of the M1 hand region showed significant recovery at around 4 months. At 9 months, inactivation of residual M1 with muscimol did not reinstate the deficit, only lesions to ventral and dorsal premotor regions did so (Liu and Rouiller, 1999). This is a very important result as it shows that recovery was mediated not by a non-hand territory of M1 reorganising to become a hand territory, but instead by premotor regions with *pre-existing* hand representations (McNeal et al., 2010). This conclusion is further strengthened by a longitudinal study of motor recovery in patients after stroke, which found that despite significant recovery there were no significant changes in fMRI markers of either task-evoked brain activity or cortico-cortical connectivity (Figure 10E–G; Branscheidt et al., 2022; Ejaz et al., 2018). This and several other studies challenge the notion of any substantial cortical reorganisation after stroke (see Krakauer and Carmichael, 2017, for comprehensive review).

Given all these results, a more plausible explanation is that motor recovery is enabled by upregulation, or strengthening, of residual descending pathways from cortex to the brainstem and spinal cord (Lin et al., 2018; Starkey et al., 2012; Wahl et al., 2014). While these connections are always present, they might be sparse and weak, but nevertheless are labelled as ‘hand’ pathways in the baseline stimulation-defined map. But following brain damage, they may become facilitated and thus aid restoration of motor function. It is possible that the observed temporary map expansion following intensive post-stroke rehabilitation might reflect the process of facilitating these residual pathways. Therefore, instead of functional reorganisation, a more parsimonious conclusion is that map expansions and contractions are epiphenomenal to the neural basis of the improved motor performance. This conclusion is consistent with the above-mentioned studies of S1 finger maps, showing that the relevance of cortical map changes to sensory abilities are minimal. It is problematic that these original motor recovery studies in monkeys are cited frequently in support of the notion of reorganisation when closer scrutiny does not support this conclusion.

### Can the cortical body representation incorporate tools and prosthetic limbs?

Is it possible that the examples we’ve reviewed so far have failed to meet our criteria for reorganisation, namely an area that assumes a qualitatively new functional identity, because of our focus on sensory and motor processing? Indeed, the majority of cases we’ve considered so far concern modular remapping, where the local topographic organisation might be too limiting to afford a new functional architecture. In this final section, we consider an example of representational changes that have been ascribed to our brain’s ability to adapt to new circumstances dynamically and flexibly, without profound peripheral or central change. Specifically, we consider the popular view that our brain can incorporate inanimate objects into our body models (or schemas).

It is often suggested that even in everyday situations, for example, when people drive or use a pencil, they can experience an alteration of their body representation. Specifically, the flexible nature of the body representation allows instruments to become incorporated into it (Ianì, 2021). When we drive the car, we can ‘feel’ our peripersonal space expanding to engulf the car; When we write with the pencil we can ‘sense’ the paper we are writing on. In their famous 1911 lecture before the Royal College of Physicians, Head and Holmes suggested that our cortical body representation can incorporate any object that is relevant for our movements, such that a woman may extend her cortical body model to incorporate the feather on her hat (Head and Holmes, 1911). This action-oriented representation, and in particular the idea that our cortical body representation can be extended to incorporate external objects that are relevant to our motor repertoire, has stimulated much interest into the neural representation of hand-held tools.

While theoretically appealing, there is little direct evidence for tool incorporation at the neural level. The most famous study to support the idea that our body representation can dynamically incorporate hand-held tools was conducted by Iriki et al., 1996. This study built on a well-described phenomenon, where some neurons within higher-order somatosensory areas (e.g. intraparietal cortex [Colby and Duhamel, 1991] and Broadman area 7b [Leinonen and Nyman, 1979]) also respond to visual information. An important feature of these bimodal neurons is that they tend to respond to visual stimuli moving near the tactile receptive field (on the face, arm, or hand) – but not far away from it. These neurons provided an elegant testcase for the tool-use embodiment hypothesis. If, while using the tool, the object integrates with the sensory representation of the arm, then arm-centred bimodal neurons should now respond to visual information presented out of arm’s reach but within the tool’s reach.

**Figure 11.**
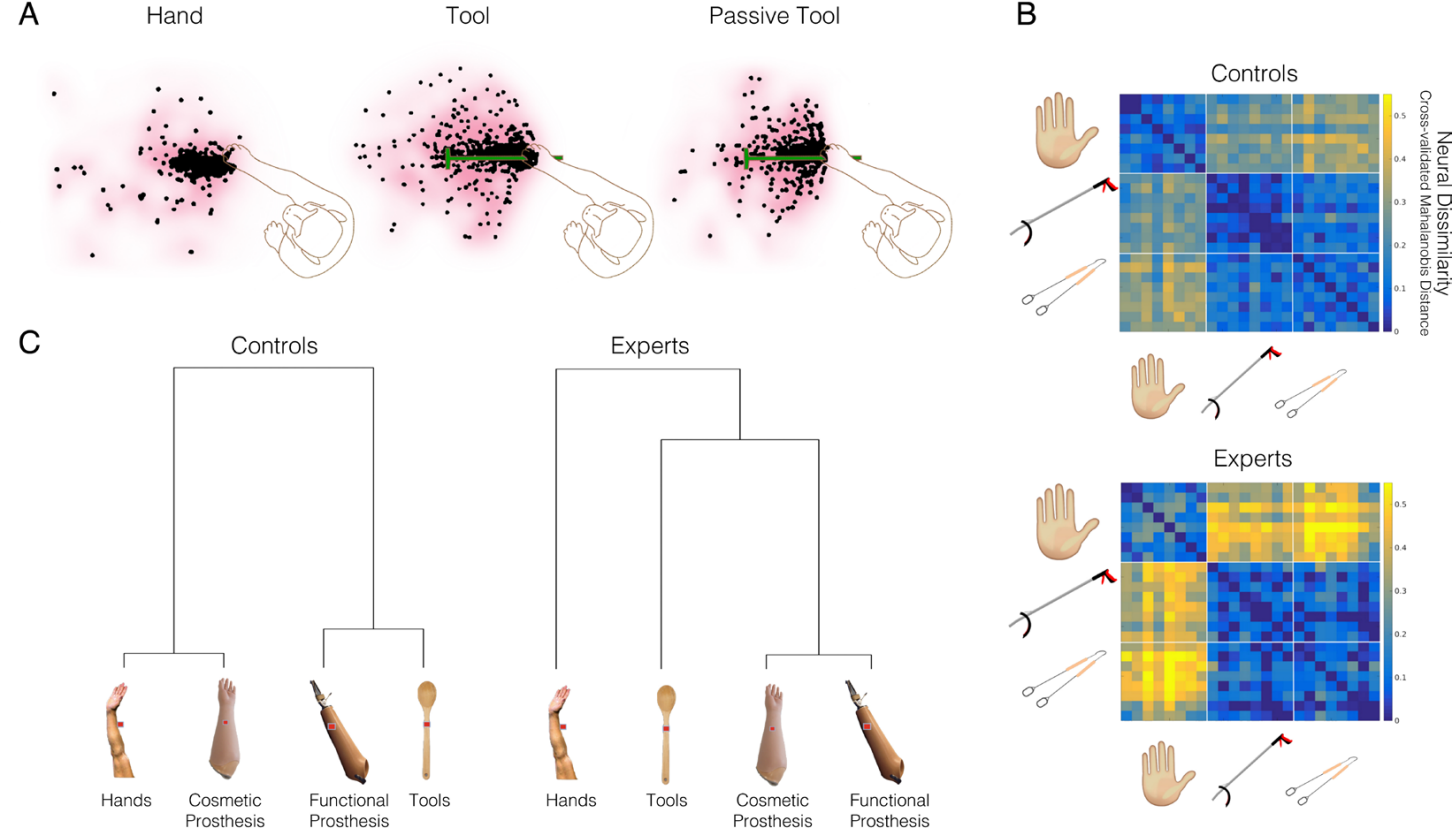
Tool-use is not associated with increased embodiment. (**A**) An example neuron from the famous study by Iriki and colleagues, showing spatially expanded ‘visual’ responses after the monkey used a tool (middle panel), relative to hand use (left) or passive holding of the tool (right). The dots show the spatial positions where food pieces, moved towards and away from the monkey, elicited neural responses. Qualitatively, the middle ‘Tool’ condition triggers more neural responses further away from the monkey’s hand. This has been taken as evidence that the tool becomes incorporated into the body representation of the monkey. (**B–C**) An arguably more direct means to establish the link between tool and hand representation is by comparing between them. (**B**) Prosthetic limbs are assistive devices designed to substitute the missing hand function, therefore providing a strong test case for researching embodiment. A linkage tree showing fMRI representational similarities between hands, tools, and two prosthesis types in the extrastriate body area (occipitotemporal cortex) of controls (Controls; left) and 32 prosthesis users with a missing hand (Experts; right). Longer branches indicate greater dissimilarity across categorical representations. Controls tend to represent prostheses similarly to either hands or tools, based on their visual (dis)similarity to biological upper limbs. The expert prosthesis users visually represent both prosthesis types more similarly to each other, and less like a hand (or a tool). (C) London litterpickers routinely use a gripper tool as part of their job, making them expert tool users. fMRI representational dissimilarities across various video clips showing object manipulation with hands, an expert tool (litterpicker), and a non-expert hand-held tool (thongs). Warmer colours show greater dissimilarities across categorical representations. Relative to novices, experts show greater neural dissimilarity (more distinct) representation of the tools compared with hands. Images modified from: (A) is adapted from Figure 1 from Maravita and Iriki, 2004; (B) is reproduced from Figure 2 from Schone et al., 2021; (C) is reproduced from Figure from Maimon-Mor and Makin, 2020.

Two monkeys were trained to retrieve food pellets using a rake. The monkey will first seize the food pellet with the tip of the rake and pull the pellet closer to retrieve it with their other hand. Once trained, the researchers recorded visual responses in the anterior bank of the intraparietal cortex – corresponding to Broadman Areas 2 and 5. These are considered mid-to-higher-order somatosensory areas, though it is important to point here that bimodal neurons are rare in the regions Iriki was recording from (e.g. 6%, as reported by Graziano et al., 2000). In this seminal study, the authors noted that while training the monkeys, some hand-centred tactile neurons had developed visual properties, which spatially aligned with the inherent tactile receptive fields. To test those visual responses, the researchers held pieces of food pellets that were moved towards (and away from) the hand. The experimenters logged the spatial position at which the bimodal neuron became responsive (Figure 11A). They noticed that, after the monkey used the tool for a short period to retrieve food, some visual responses were initiated from a greater distance, compared to after the monkey retrieved food with their own hand (middle versus left images in Figure 11A). What’s more, the expansion of visual responses was not found after the monkey was made to hold the tool passively (without using it to retrieve food). Therefore, the modulation of the bimodal neuron has been interpreted as reflecting the incorporation of the tool into the body representation of the monkey, based on their action intent.

This hugely influential study has solidified the idea that using tools leads to integration between the neural representations of the body and the tool, a process known as embodiment (Maravita and Iriki, 2004). Embodiment has been suggested to promote intuitive control, learning, and comfort when using a tool. Therefore, biomedical and robotics engineers are interested in harnessing embodiment as a mechanism to enable technology adoption and successful rehabilitation (Page et al., 2018; Preatoni et al., 2021; Rognini et al., 2019; Valle et al., 2018; Zbinden et al., 2022). However, considering the insurmountable confounds in the experimental design detailed above, one must wonder – does the study truly imply expansion of hand-centred visual responses, rather than, say the predictive value of the piece of food (Kandula et al., 2015)? Or even a somatosensory response – either anticipatory, or due to some motor responses made by the monkey? (while the monkey’s hand was immobilised, more subtle muscle response was not recorded). Indeed, when the pieces of food were replaced by a non-edible bait, the visual responses disappeared. What’s more, while the prediction outlined before provided very specific outcome measures, relating to the reaching distance afforded by the tool, relative to the arm – no attempt of precise quantification of the changes to receptive field properties was made.

A conceptual problem with the Iriki study, and many others endeavouring to replicate it (for a critical review see Holmes, 2012), is that the ‘visual’ measure used here was indirect – it does not capture the representation of the tool itself, or even of the body, but rather a neural proxy of the body (peripersonal space). This is why it is so easy to pick this study apart – the modulated response could mean anything, from differences in low-level signals to top-down modulation of arousal and attention. A more recent approach, using electroencephalography, examined how tactile information carried by a hand-held stick is processed by the somatosensory system as compared to the hand itself (Miller et al., 2019). The authors found that cortical processing of location is similar for tools and the body, suggesting that the two may be processed equivalently in the cortex. But is this a surprising finding, considering the sensory information from the hand-held tool is mediated by the mechanoreceptors on the hand? Rather than this low-level representation being attributed by the nervous system to the tool, it is more likely that it is attributed to the mechanoreceptors in the hand that mediate this information. Considering the study was done when participants were passively gripping a stick, which they did not previously use as a tool, this begs the question – did this study capture anything profound about tool-use? Other studies, directly comparing the representation of an expert tool (a litterpicker in street cleaners [Schone et al., 2021]; prosthetic limbs in amputees [Maimon-Mor and Makin, 2020]) using fMRI representational similarity analysis in fact demonstrate the opposite effect – experts do not integrate but rather differentiate their tool representation from a biological hand representation (Figure 11B and C).

It is important to note that these latter studies relied on visual representation of the hand and tool, and as such the sensorimotor system might develop diverging templates to represent the tool. Nevertheless, while it counters street ‘wisdom’, the idea that expert tools are represented *separately* from our hands makes a lot of sense. There are little similarities in the low-level features of tools, relative to hands. Even relatively simple tools (such as a pencil) also require association to higher-order cognitive resources (such as language). Rather than conforming to the neural body representation to create these broader associations and contain the tool-related inputs, it might be more efficient for the brain to develop a distinct representation of these tool-related processes to better guide motor control (Makin et al., 2020). This is truer for expert tool-use, which is guided and refined by learning. Finally, if our body representation were to be so flexible as to allow any object that is relevant for our actions (such as the feather on the hat from Head and Holmes’ example) to be incorporated into the body representation, one must wonder if body representation would be as robust and reproducible as it is across individuals. Going back to our original example from the introduction, we all share very similar cortical functional organisation despite our unique life experiences.

Then what about the clear and vivid intuition that we have, that when we use a tool, it feels like an extension of our own body? While introspection might provide viable routes to forming scientific hypotheses, we hold that evidence should prevail. There is no strong scientific evidence to support this idea.

### Summary and conclusions

Here, we have reviewed several of the seminal studies that led to the strong notion of brain reorganisation – a qualitative switch in the functional identity or computational capacity of a given brain area – as a mechanism for driving physiological and behavioural change after various forms of insult to the nervous system. To aid our review, we set out three necessary criteria for determining reorganisation. First, the ‘reorganised’ area is expected to respond to a novel input (or produce a novel output, in the case of M1). Second, the local computational capacity of the ‘reorganised’ area should change. Finally, this local processing should be coherently read out as a novel function to guide behaviour, meaning a change in the area’s connectional fingerprint. We conclude that none of the canonical studies we reviewed convincingly fulfil these criteria. As such, we did not find good evidence for the existence of functional pluripotency for cortex in any period of development; instead, the assignment of brain function to a given cortical structure is likely to be largely fixed at birth. Cochlear implants for congenital deafness, sight restoration for congenital blindness, and peripheral or central sensory stimulation in amputees/deafferented patients, all point to functional preservation of native processing in cortical territories long deprived of their natural inputs; they have not been ‘taken over’ by an alternative function. There are three points that recur throughout this piece: (i) There is no qualitative ‘take-over’ of function in primary sensory and motor cortices. (ii) Map expansion effects in these cortical areas reflect unmasking of *pre-existing* inputs, usually of adjacent representations. (iii) Upregulation of modality-agnostic association areas can be mistaken for reorganisation because these areas start responding more strongly to a second modality. An example of this was given in the section on cross-modal plasticity in congenitally blind individuals: extra-striate cortex can process auditory input, but crucially this is also the case in the sighted, indicating that the region is *already* input modality invariant, to a degree. The impressive behavioural phenomena seen in the various clinical cases that we have discussed are attributable to learning-related recruitment of higher-level cortical computations that can be functionally re-focused on residual primary area representations. These higher-level computations occur in association areas that can be adjacent to primary modality areas or distant, for example, in their homologs in the opposite hemisphere, as is the case for language recovery after perinatal stroke. In all cases, there is no need to invoke changes (reorganisation) in the representations in primary areas themselves.

Why then, given the difficulty of finding convincing evidence for cortical reorganisation, is cortical reorganisation such a widespread and accepted idea in contemporary neuroscience, neuroengineering, and clinical rehabilitation? As we discuss throughout this piece, we think a key culprit for this conceptual confusion is an artefact: categorical cortical maps in primary areas. Textbooks (and more recently – the internet) are littered with colourful brain maps, ascribing brain function based on sharply demarcated boundaries. For example, in the primary somatosensory cortex, the brain is divided into a map of distinct body parts. Such maps are derived by a winner-takes-all thresholding process whereby the stimulus or condition that evokes the highest activity in the experimental paradigm leads to only one inferred function being ascribed to the whole area. In addition, the ‘winner’ function itself is somewhat arbitrary as it is determined only with respect to the set of conditions chosen for it to compete against. In any event, what is just a weighted preference for a given feature, as measured by activity gain, is turned into all-or-none categorical dominance. But as highlighted by Penfield himself, the representation of different body parts is highly distributed and boundaries are fuzzy at best (Catani, 2017). In other words, the organisation that is imposed by these human-made maps may not fully capture the underlying functional organisation.

Another consequence of an over-emphasis on maps with sharp borders is the wrongful interpretation of their expansion into adjacent areas as evidence for reorganisation. This is often described as ‘remapping’, ‘remodelling’, ‘expansion’, ‘rewiring’, ‘take-over’, ‘invasion’, ‘reoccupation’, and even ‘capture’ (Merzenich et al., 1983a; Merzenich et al., 1983b). As we discuss throughout the piece, it is essential that we do not turn the result of a mode of analysis – remapping – into a true phenomenon (reorganisation). Instead, as we have seen, a change to map boundaries can be the consequence of learning-related strengthening of pre-existing dedicated architecture. More importantly, this ‘remapping’, regardless of the neural process it reflects, is often not causally related to behavioural recovery at all. Thus, de-emphasising brain maps with sharp borders can lead the field away from positing a special form of plasticity predicated on qualitative changes in the computational capacity of an area. Instead, a given brain area may have the capacity to receive information from more diverse sources than is suggested from the derivation of discrete maps. How these more diverse inputs are processed will of course be constrained by the brain area’s local and global connectivity. This connectivity fingerprint pre-determines to a large degree what the local computational capacities of a given brain area are, and how these computations are read out by downstream areas to inform behaviour. Even when inputs are engineered by scientists and detoured into a different brain area, as discussed in the section ‘Experimentally induced rewiring of retinal input in newborn ferrets’, successful processing of this rewired input depends on the recipient architecture *already* being compatible with the novel input.

For illustration, let us reconsider the instructive example of cross-modal plasticity in congenitally blind individuals. Based on Figure 5, one can infer that receptivity to auditory input already exists in the occipital cortex of sighted participants. The reduced information content observed for auditory inputs in the sighted brain may be due to masking of the weak auditory input by the stronger visual input. Nevertheless, this latent auditory information might still be processed in the same way in sighted individuals. ‘Passive’ plasticity mechanisms, such as unmasking and homeostatic plasticity, are all that need to be invoked to explain the observed ‘cross-modal plasticity’. In addition to these passive processes, practice effects can improve how this typically latent capacity can become more functionally effective than it is in sighted individuals. Indeed, as we illustrate in Figure 1, top-down contextual information may also amplify this training effect. It is also likely that critical periods in development are more conducive to such training effects. Consequently, a variety of mechanisms can be posited to explain how a visual brain area can become functionally more responsive to auditory input without the need to resort to the concept of reorganisation. In our revised framework, any opportunities for functional change throughout life are limited to a pre-existing structural ‘blueprint’ via Hebbian and homeostatic plasticity mechanisms.

An important implication of shifting from an emphasis on reorganisation to one on training-guided neuroplasticity is that neurorehabilitation strategies should switch from trying to induce cortical reorganisation and instead take better advantage of existing residual anatomy. For example, spinal cord stimulation can be used to enhance the functionality of the residual corticospinal tract in both stroke and spinal cord injury (Angeli et al., 2018; Harkema et al., 2011; Powell et al., 2022). This approach is entirely about upregulation of residual architecture and not about reorganising alternative cortical regions. Critically, these approaches have been shown to benefit from combining them with intense and high-dose training protocols (Angeli et al., 2018; Harkema et al., 2011). The brain’s striving for stability despite dramatic input/output changes also offers an arguably more secure platform for developing novel neuroengineering technologies for substitution. The realisation of neuroprostheses, a set of technologies designed to artificially restore lost sensory and motor capabilities by directly interfacing with the peripheral or central nervous system, critically depends on some degree of preservation of the original sensory and motor pathways. Augmentation technologies, on the other hand, attempt to reallocate sensory and motor pathways to enhance our sensory and motor abilities (Dominijanni et al., 2021), and it is important to appreciate that opportunities in this regard may be more limited than previously envisaged and require extensive training (Makin et al., 2023).

Finally, it is important to greatly increase our understanding of how critical periods enhance responsivity to training. There is no doubt that opportunities for plasticity are enhanced in early development. However, the factor that makes a difference with age is not the loss of some pluripotent cortical capacity that allows reorganisation. Moreover, critical periods need not be restricted to early life. For example, research in stroke patients suggested that ischemia creates conditions that are favourable for plasticity and have even been referred to as a second sensitive period (Zeiler and Krakauer, 2013). Similarly, it has been suggested that sensory deprivation can also promote plasticity and learning (Dempsey-Jones et al., 2019). Rehabilitation will likely be most effective within these limited windows of enhanced plasticity (Dromerick et al., 2021; Krakauer et al., 2021). Complete explanations for the differential responsiveness of a pre-dedicated cortical territory to training-related upregulation during certain time periods are still lacking. However, finding them is more likely if one does not couch the question in terms of cortical areas switching from having pluripotency to not having it.
